# Boronate Ester Hydrogels for Biomedical Applications: Challenges and Opportunities

**DOI:** 10.1021/acs.chemmater.4c00507

**Published:** 2024-07-09

**Authors:** Léa Terriac, Jean-Jacques Helesbeux, Yves Maugars, Jérôme Guicheux, Mark W. Tibbitt, Vianney Delplace

**Affiliations:** † Nantes Université, Oniris, CHU Nantes, INSERM, Regenerative Medicine and Skeleton, RMeS, UMR 1229, F-44000 Nantes, France; ‡ Univ Angers, SONAS, SFR QUASAV, F-49000 Angers, France; § Macromolecular Engineering Laboratory, Department of Mechanical and Process Engineering, ETH Zurich, 8092 Zurich, Switzerland

## Abstract

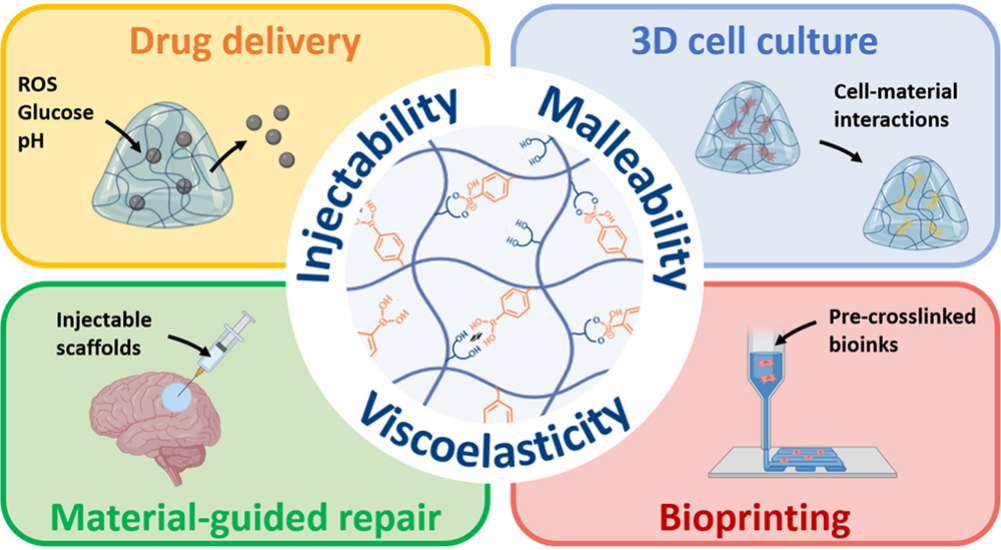

Boronate ester (BE) hydrogels are increasingly used for biomedical applications. The dynamic nature of these molecular networks enables bond rearrangement, which is associated with viscoelasticity, injectability, printability, and self-healing, among other properties. BEs are also sensitive to pH, redox reactions, and the presence of sugars, which is useful for the design of stimuli-responsive materials. Together, BE hydrogels are interesting scaffolds for use in drug delivery, 3D cell culture, and biofabrication. However, designing stable BE hydrogels at physiological pH (≈7.4) remains a challenge, which is hindering their development and biomedical application. In this context, advanced chemical insights into BE chemistry are being used to design new molecular solutions for material fabrication. This review article summarizes the state of the art in BE hydrogel design for biomedical applications with a focus on the materials chemistry of this class of materials. First, we discuss updated knowledge in BE chemistry including details on the molecular mechanisms associated with BE formation and breakage. Then, we discuss BE hydrogel formation at physiological pH, with an overview of the main systems reported to date along with new perspectives. A last section covers several prominent biomedical applications of BE hydrogels, including drug delivery, 3D cell culture, and bioprinting, with critical insights on the design relevance, limitations and potential.

## 1 Introduction

Dynamic hydrogels are increasingly used for biomedical applications.^1−3^ Dynamic hydrogels are hydrated molecular networks that formed via reversible molecular interactions. The dynamic nature of these molecular networks is governed by chemical and thermodynamic equilibria and results in tunable viscoelasticity, which is the property of a material to exhibit both viscous and elastic behavior. When properly adjusted, bond rearrangement and the associated viscoelasticity in dynamic hydrogels enables advanced processing properties, such as injectability, printability, and malleability, which are critical for many biomedical applications.^4^ Viscoelasticity is also an essential characteristic of the extracellular matrix,^5^ and viscoelastic hydrogels find applications in 3D cell culture, where they better reproduce natural cell–material interactions.^6,7^

To obtain dynamic hydrogels, various reversible molecular interactions can be used, including ionic interactions, hydrophobic–hydrophobic interactions, supramolecular interactions, and dynamic covalent reactions.^8^ Dynamic covalent reactions are reactions that produce reversible covalent bonds. They present the advantage of being based on small and affordable molecules that can be grafted easily to many polymers for subsequent cross-linking. The structure of these reactive molecules can often be adjusted to tune the chemical equilibria of the dynamic covalent reactions and, in turn, tune the physical properties of the dynamic hydrogels. The most common dynamic covalent reactions used in the design of dynamic hydrogels are the Diels–Alder reaction,^9^ Schiff Base formation,^10^ and boronate ester (BE) formation.^11^

BE is the reversible product of the reaction between a boronic acid derivative and a diol. The formation of BEs has been studied extensively for the column-based purification of natural polyols, such as monosaccharides, and, more recently, BE formation is attracting increasing attention as a chemical tool for dynamic hydrogel design.^11^ Like other dynamic hydrogels, BE hydrogels exhibit viscoelasticity, injectability, and malleability, among other useful properties. More importantly, various natural molecules (i.e., sugars) can compete with diols for BE formation, and boronic acid derivatives are sensitive to redox reactions, together making BE hydrogels highly sensitive to their environment, which is useful for the design of stimuli-responsive materials.^12^ However, BE formation is favored at basic pH, and designing BE hydrogels at physiological pH (≈7.4) is challenging. While this has constrained the development of BE hydrogels for biomedical applications, the scientific community is now taking a fresh look at this exciting chemical challenge, offering new molecular solutions and perspectives.

This review article summarizes the state of the art in BE hydrogel design for biomedical applications. First, we discuss updated knowledge in BE chemistry, with details regarding molecular mechanisms and new approaches for BE formation at physiological pH. This fundamental discussion serves as a solid basis of understanding to the second section that is dedicated to BE hydrogel design, where we provide a critical overview of several prominent systems developed to date as well as new perspectives. The third section covers the main biomedical applications of BE hydrogels, from drug delivery and 3D cell culture to bioprinting.

## 2 Boronate Ester Chemistry

**Figure 1:**
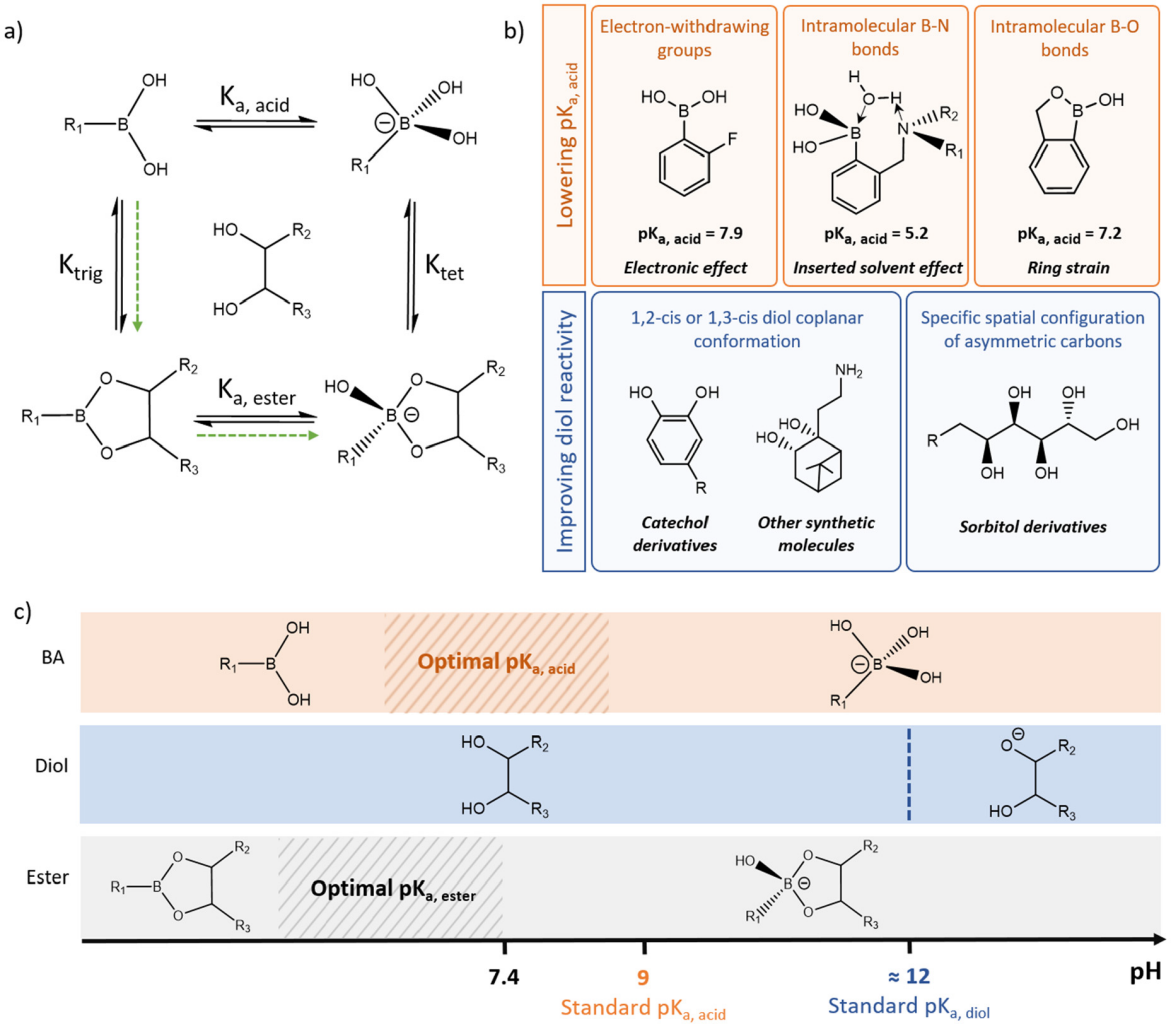
Strategies to favor BE formation at physiological pH. (a) Chemical equilibria involved in BE formation and stabilization, including the BA/boronate anion equilibrium (p*K*_a, acid_), the boronic/boronate ester equilibrium (p*K*_a, ester_), the BA/BE ester equilibrium (p*K*_trig_), and the boronate anion/BE ester equilibrium (p*K*_tet_). Green arrows indicate the favorable mechanism for bond formation, where the more reactive neutral BA reacts with a diol followed by boronic ester ionization and stabilization as a boronate ester. (b) Main strategies investigated for BE formation and stabilization at physiological pH, including the design of PBAs with lower p*K*_a, acid_ and the design of diols with improved reactivity toward PBA derivatives. (c) Schematic of the optimal range of p*K*_a, acid_ for BE formation and stability at physiological pH, using a vicinal diol with a standard p*K*_a, diol_ of ≈12. A p*K*_a, acid_ lower than 9 would favor neutral BA reactivity and lead to a p*K*_a, ester_ well below 7.4 for BE stability.

The esterification reaction between a boronic acid (BA) derivative (*R-B(OH)*_*2*_) and a diol produces a reversible BE with the loss of an H_2_O molecule (Figure 1). Due to the reversibility of the reaction, BA/diol reactivity and BE stability both have an impact on the chemical equilibrium. The three species involved in the reversible reaction, namely BA, diol, and BE, all exist under various forms. In water, the proportion of each of their forms depends on the acid dissociation constant (K_d_), the anionic-to-neutral molecule ratio (p*K*_a_), and pH. To control BE formation, one needs to carefully consider the effects of molecular structures on the chemical equilibrium and the associated reaction mechanisms.^11^

### 2.1 Mechanism of Boronate Ester Formation

BAs constitute a class of Lewis acids that include an electron-deficient boron center.^13^ In water, BAs exist in a neutral form and an anionic form that is called boronate anion.^14^ The two forms are in ionic equilibrium and the ratio of the species depends on the p*K*_a_ of the BA (p*K*_a, acid_).^15^ Typically, phenylboronic acids (PBAs), which comprise a commonly used family of BA derivatives, have a p*K*_a, acid_ of ≈9. The formation of a boronate anion through the addition of a hydroxyl group to a BA induces a change in the hybridization from trigonal (sp^2^) to tetrahedral (sp^3^). The tetrahedral hybridization stabilizes the boronate anion, which in turn makes it less reactive toward diols.^16^ Thus, ester formation occurs preferentially with the neutral BA, and pH values below the p*K*_a, acid_ favor esterification.^17^ This reactivity is characterized through two esterification equilibrium constants (K_eq_) corresponding to the combination of a diol with either a neutral BA (K_trig_) or a boronate anion (K_tet_).

Similar to BA, the ester resulting from the reaction of a BA and a diol exists both as a neutral (boronic ester) and an anionic species (BE). The two forms are in chemical equilibrium that is defined by the p*K*_a_ of the ester (p*K*_a, ester_). Upon BA esterification, the obtained neutral boronic ester has a trigonal molecular geometry that makes it prone to hydrolysis.^16^ The anionic BE has a tetragonal geometry that makes it more stable than its neutral counterpart. Thus, to favor ester stability, the p*K*_a_ of a boronic/boronate ester couple (p*K*_a, ester_) should, in theory, be as low as possible to maximize the proportion of the more stable BE at a given pH. The ester stability is directly linked to the esterification equilibrium constant (K_eq_), which provides quantitative insights on the molecular reactivity and ester stability.

The effect of the molecular charge on the equilibrium also plays a role in BE formation and stability. Forty years ago, Van Duin et al. defined the “charge rule” based on empirical data, hypothesizing that BE “shows the highest stability at that pH where the sum of charges of the free esterifying species is equal to the charge of the ester”.^18^ In this study, the authors observed that ester formation was favored at a pH value both higher than the p*K*_a_ of the diol (p*K*_a, diol_) and lower than the p*K*_a, acid_, using glycolic acid (p*K*_a, diol_ = 3.82) and boric acid (p*K*_a, acid_ = 9) as model molecules. A similar phenomenon was observed by others when combining Alizarin Red (p*K*_a, diol_ = 5.5) and PBA (p*K*_a, acid_ ≈ 9).^19^ Here, the authors suggested that this empirical observation was due to the necessary balance between species reactivity and the stability of the resulting ester. In short, increasing pH leads to an increase in the proportion of the more stable boronate ester form; however, it also decreases the fractions of the neutral and more reactive forms of BA and diol. Consequently, the authors concluded that averaging the p*K*_a, acid_ and p*K*_a, diol_ values of a given BA/diol pair would provide a good indication of the optimal pH to promote overall ester formation and stability. However, in both studies, the observations and conclusions were based on the use of hydroxycarboxylic acids and/or diols with unusually low p*K*_a, diol_ values (3.82 and 5.5, respectively), far below the p*K*_a, diol_ value of common diols, which is of ≈12. Thus, the authors studied BA/diol couples with a p*K*_a, acid_ higher than p*K*_a, diol_, which is the reverse order compared to those of common BA/diol pairs and makes the charge rule hard to generalize. While still mentioned in some articles, we strongly encourage readers to use the charge rule with extreme caution for their mechanistic investigations, especially when working with common diols.

In sum, it is now generally accepted that neutral BAs react preferentially and that the anionic BE constitutes a more stable product. While the charge rule may be controversial, we can at least hypothesize that a p*K*_a, acid_ value close to the working pH should favor BE formation and stability. This reasoning may still be too simplistic as it solely considers the effects of pH, p*K*_a, acid_, and p*K*_a, ester_ on species reactivity and stability. It also mainly focuses on the BA structure without consideration for the molecular characteristics of diols and specificities of BA/diol pairs that could enhance or hinder reactivity and product stability.

### 2.2 Strategies to Favor Boronate Ester Formation at Physiological pH

BE formation from PBAs and most of the natural diols, such as glucose or fructose (p*K*_a, diol_ ≈ 12), is favored at basic pH (i.e., pH 8–12).^14,18^ This has greatly hindered the development and use of BEs in biomedical applications where physiological pH is almost always a relevant design criterion. However, over the past 20 years, careful investigations of the effects of molecular structures on BE formation and stability have led to the identification of several BA derivatives and diols that allow for BE formation at physiological pH. In this context, two main strategies have been investigated (Figure 1b and c): *i*. designing PBAs with lower p*K*_a, acid_ in order to lower the p*K*_a, ester_ and favor BE stability; and *ii*. designing new diols with improved reactivity toward PBA.

#### 2.2.1 Designing PBA Derivatives with Low p*K*_a, acid_

The p*K*_a, acid_ of standard PBA (≈ 9) favors the presence of the more reactive neutral form of PBA at pH 7.4. Thus, PBA reactivity is not a challenge per se at physiological pH, and limited binding is believed to result primarily due to the poor stability of the neutral boronic ester. The p*K*_a, ester_ is intrinsically linked to the p*K*_a, acid_. It is also, by definition, lower than the p*K*_a, acid_ because the formed ester is more prone to ionization due to ring strain.^20^ As such, it has been suggested that designing PBAs with lower p*K*_a, acid_ values would maintain a sufficient amount of the reactive PBAs while shifting the p*K*_a, ester_ toward lower values to favor ester ionization and stabilization at physiological pH. To lower the p*K*_a, acid_ of PBA derivatives, three main strategies have been considered: the addition of electron-withdrawing groups, the use of an intramolecular B–O bond, and the use of an intramolecular B–N bond.

The addition of an electron-withdrawing group on PBA often increases the acidity of the PBA by increasing the boron electrophilicity, facilitating sp^3^ hybridization and lowering its p*K*_a, acid_.^16^ A greater influence of the electron-withdrawing group is expected in the ortho and para positions. Nitro compounds (NO_2_),^21^ fluor,^22,23^ amide,^24,25^ carboxyl,^26^ formyl,^25^ carbamoyl^25^ and cyano^27^ groups have been used as electron-withdrawing groups to favor BE formation at a lower pH. For example, 2-formyl-PBA exhibited a p*K*_a, acid_ of 7.5, leading to an equilibrium constant with glucose at physiological pH that is five times greater than that of PBA (K_eq_ of 22 M^–1^ and 4.6 M^–1^, respectively).^20,25^ Similarly, 4-(methylcarbamoyl)-PBA with a p*K*_a, acid_ of 7.9 has a binding constant with glucose at physiological pH that is two times greater than that of PBA (K_eq_ of 8.8 M^–1^ and 4.6 M^–1^, respectively).^20,25^ The introduction of a nitrogen within the PBA ring, under the form of a 3-pyridinylboronic acid (PyBA), further lowers the p*K*_a, acid_ value to ≈4.4, and PyBA exhibits a relatively high binding constant with glucose at pH 7.4 (164.7 M^–1^).^28^

The intramolecular coordination between the boron atom and a proximal amine was found to decrease the p*K*_a, acid_ of PBA. The addition of an *ortho*-aminomethyl group in PBAs has long been studied. In the 1960s, seeking a strategy to synthesize stable poly(boronate esters), François and Clément first demonstrated that an *ortho*-aminomethyl-PBA (*o*-AM-PBA), which they described as a “scorpio-type” molecule at the time, better resisted hydrolysis.^29,30^ Decades later, in the context of molecular templating, Wulff and co-workers further described the improved reactivity of *o*-AM-PBA toward sugars (i.e., natural polyols), confirming a much lower p*K*_a, acid_ value of ≈5.2.^31,32^ Beyond its lower p*K*_a, acid_, the actual binding mechanism of *o*-AM-PBA, sometimes referred to as Wulff-type PBA,^32^ has long been debated. Indeed, it was first postulated that the lower p*K*_a, acid_ resulted from the formation of a B–N covalent bond.^33,34^ However, it is now believed that it results from a specific mechanism where a solvent molecule is temporarily inserted between the boron center and the nitrogen of the *o*-aminomethyl group.^35^ At neutral pH, the field effect from the ammonium cation would facilitate the insertion of a water molecule, leading to boron sp^3^ hybridization. The inserted molecule would then be temporarily lost, producing a sp^2^-hybridized intermediate that is highly reactive toward diols. This phenomenon was referred to as the “loose-bolt effect” in the specific context of fluorescence imaging where the fluorescence signal of specific PBA derivatives is quenched by a molecular vibrating effect linked to the inserted solvent mechanism.^35^ The loose-bolt metaphor is less relevant in other contexts, and the term “inserted solvent effect” is preferred. This specific mechanism may explain the remarkable reactivity of *o*-AM-PBA toward diols at physiological pH despite a prohibitively low p*K*_a, acid_ value, i.e., low amount of reactive BA for esterification. However, the few reported binding constants of *o*-AM-PBA are surprisingly low, with a K_eq_ of 380 M^–1^ with sorbitol at pH 7.4 comparable to the one of PBA (370 M^–1^),^20,25^ which is in contradiction with the observed high reactivity of *o*-AM-PBA and calls for further investigation. Overall, the low p*K*_a, acid_ of *o*-AM-PBA and the unique reactivity of *o*-AM-PBA make it particularly attractive for use at physiological pH. For a more detailed understanding of the mechanism of BE formation with *o*-AM-PBA, the readers can refer to the excellent review article by Sun et al.^36^

In 2006, Dowlut et al. mentioned for the first time the *o*-hydroxymethyl-PBA, with binding capacity to fructose (K_eq_ of 606 M^–1^) higher than that of PBA (K_eq_ of 79 M^–1^) at pH 7.4.^34^ They expanded this study with the evaluation of *ortho*-substituted PBAs, and other PBA derivatives, binding with glycopyranosides and reported a uniquely higher binding for *o*-hydroxymethyl-PBA at pH 7.4.^37^ Taken together, these studies highlighted the unusual capacity of this new class of PBA to bind diols at physiological pH, which was attributed to the presence of an intramolecular B–O bond that induces ring strain on the neutral form of PBA, thus significantly lowering its p*K*_a, acid_.^38^ This intramolecular B–O bond can be obtained by modifying a PBA with an *ortho*-hydroxymethyl group, then called benzoxaborole (BX), or with an *ortho*-hydroxyethyl group, then called benzoxaborin.^38^ The relatively low p*K*_a, acid_ value of BX (7.34) compared to benzoxaborin (8.59) leads to higher binding constants with diols (typically, with fructose: 461 M^–1^ and 87 M^–1^, respectively), making benzoxaborin less interesting.^39^ Recently, a new molecule with an intramolecular B–O bond, namely the 4-dihydro-2H-benzo[e][1,2]oxaborinin-2-ol (1,2-BORIN), was reported to have a p*K*_a, acid_ of 6.2 and a binding constant with fructose of 531 M^–1^ at pH 7.4, making it a promising alternative to BX.^39^

#### 2.2.2 Designing Diols with Improved Reactivity

Diol reactivity toward PBAs also plays an important role in BE formation. This parameter has long been investigated; however, most of the work was done in the context of natural polyol and diol purification. Thus, current knowledge regarding diol reactivity toward PBAs is mainly based on experiments conducted on natural molecules (e.g., monosaccharides). Experiments have demonstrated that the presence of 1,2- or 1,3-cis diols in a coplanar conformation favors reactivity toward PBAs.^40^ However, cis diols rarely occur in a locked conformation in natural molecules. For example, natural monosaccharides, which are natural polyols, rarely exist as coplanar cis diols. Monosaccharides exist in the form of two cyclic hemiacetals in equilibrium: furanoses (5membered rings) and pyranoses (6-membered rings). Cis diols are mainly present on furanoses, which usually account for a small percentage of total hemiacetals (≈ 0.14 to 30%), leading to limited reactivity toward PBA.^41,42^ Typically, glucose has a low occurrence of furanoses (0.14%), and only weakly binds PBA at physiological pH (4.6 M^–1^).^20,43^ Among the common monosaccharides, fructose, which has the highest occurrence of the furanose form (≈ 30%), has the highest affinity toward PBA (106 M^–1^).^20,41,43^

Catechol derivatives comprise two hydroxyl groups in a locked coplanar conformation due to their aromatic ring. This conformation, possibly along with aromaticity, confers catechol derivatives one of the highest binding constants to PBA ever reported (K_eq, catechol_ = 830 M^–1^).^20^ Thus, catechol derivatives, such as dopamine, are frequently used to form BE at physiological pH more effectively.^44−46^ However, catechols are prone to oxidation,^47,48^ limiting the ester stability over time. To overcome this limitation, a stable 1,2-cis diol was synthesized from nopol, a bicyclic alkene derived from β-pinene.^49^ The authors showed that this 1,2-cis diol, called nopoldiol, exhibited an unusually high binding constant with PBAs (typically, 27,000 M^–1^ with fluoro-PBA).^50^ Although the high binding capacity of nopoldiol is now well established, the binding constants were calculated from an NMR study performed in a mixed solvent (D_2_O/CD_3_CN, 65:35) which does not allow the comparison with PBA/diol binding constants obtained in water or phosphate buffer. To date, nopoldiol is the only non-natural 1,2-cis diol with a locked coplanar conformation reported in the literature, motivating further developments in this direction.

To increase binding affinity, sorbitol derivatives have attracted particular attention. Indeed, among the sugar derivatives, sorbitol has a relatively high binding affinity toward PBA (370 M^–1^).^20^ The sorbitol binding mechanism was investigated by ^1^H, ^13^C, and ^11^B NMR, using sorbitol and a specific PBA ((S,S)-2-(N,N-dimethyl-1-aminoethyl)ferrocene-boronic acid). This study suggested that the high binding capacity of sorbitol results from a specific spatial configuration of its asymmetric carbons that allows for tridentate binding. More precisely, two of its hydroxyl groups (on C2 and C3) constitute a primary binding site for BE formation, while a third hydroxyl group (on C5) can further bind to the boron center to stabilize the BE under a tetrahedral geometry.^51^ One limitation of sorbitol is that it does not have a specific functional group for chemical modification and grafting. Thus, three molecules with a sequence of asymmetric carbons similar to sorbitol and possessing a functional group have been used more frequently, namely gluconic acid (often obtained from gluconolactone), gluconamide, and glucamine. In various contexts, gluconic acid immobilized to other molecules via amidation and gluconamide was shown to bind efficiently to BX (477 M^–1^),^52^*o*-AM-PBA (240 M^–1^),^53^ 3-amino-PBA (1,751 M^–1^),^52^ and fluoroPBA^23^ at physiological pH. Using an indirect measurement for binding (i.e., hydrogel formation), we recently found that glucamine immobilized via an amide bond to a polymer has a specifically high binding affinity toward *o*-AM-PBA.^54^ We also showed that dulcitolamine and iditolamine, which both differ from glucamine by the configuration of a single asymmetric carbon (C4 and C5, respectively), have a much lower binding capacity. Our results suggest a more complex interaction between sorbitol derivatives and PBAs than previously thought. They also reinforce the idea of a specific binding sequence and suggest that the C4 configuration may also play a role in the binding of sorbitol derivatives. Deeper investigation of sorbitol/PBA interactions is needed, including the computational modeling of the reactivity of various sorbitol derivatives toward PBA, *o*-AM-PBA, and BX. The particular case of sorbitol also suggests that it may be possible to design specific PBA/diol interactions for enhanced binding and/or tailored applications.

Overall, the molecular structures of PBAs and diols dictate their reactivity and play a role in BE stability, together governing the feasibility of obtaining BE at physiological pH. A well-thought-out design of the PBA/diol couple is thus necessary to obtain BE-based hydrogels at physiological pH for biomedical applications.

## 3 Boronate Ester Hydrogels at Physiological pH

The dynamic nature of BE cross-linking produces viscoelastic hydrogels, i.e., materials with time-dependent mechanical properties under loading or deformation. They have advantageous properties, including self-healing, injectability, malleability, and stress relaxation. These properties are of particular interest for numerous biomedical applications, which also generally require that hydrogels form at physiological pH. As described above, BE formation is favored at basic pH, and BE hydrogels can, thus, be obtained easily under alkaline conditions without particular chemical considerations.^55−58^ For example, a monosaccharide that carries at least two diols, such as glucose, can be used to cross-link PBA-modified polymers and form BE hydrogels;^59^ however, gelation is only possible at basic pH due to the limited reactivity of glucose toward PBA derivatives. Similarly, polysaccharides such as alginate and hyaluronic acid (HA), which possess repeated diol groups, can be modified with PBA to produce single-component BE hydrogels at basic pH.^60,61^ From these studies, it is clear that the diol conformation on polysaccharides does not allow them to spontaneously form hydrogels with PBA-modified polymers at physiological pH. Overall, forming BE hydrogels at physiological pH is challenging and requires advanced PBA/diol design. Over the past 10 years, material chemists have worked on translating the knowledge of BE chemistry into effective BE cross-linking strategies at physiological pH.

### 3.1 Chemical Design for Boronate Ester Cross-Linking

For BE hydrogel synthesis, the general approach consists in mixing a PBA-containing polymer with a diol-containing polymer. Therefore, hydrogel formation and properties are highly dependent on the molecular structure of the PBA/diol pair as well as physicochemical parameters, including the degrees of substitution, polymer content, and PBA:diol molar ratio, which all need to be considered carefully.

#### 3.1.1 BE Hydrogel Design with Conventional Molecules

To obtain BE hydrogels at physiological pH in a straightforward manner, poly(vinyl alcohol) (PVA) has been combined with various PBA-modified polymers.^62,63^ Indeed, PVA is the only commercially available, synthetic polymer to possess repeated diol groups. Although its freely rotating 1,3-diol units do not theoretically favor BE formation, the large number of diol groups on PVA (high functionality) enables gelation at physiological pH with a variety of PBA-modified polymers. For example, PVA formed BE hydrogels that were self-healing, injectable, and cytocompatible when mixed with PBA-modified HA.^64^ In another study, Ishihara et al. developed another hydrogel based on PVA by combining it with a PBA-containing vinyl copolymer.^67−71^ The authors used this system to design microspheres for efficient single cell encapsulation as well as a multilayer scaffold for coculture experiments, demonstrating the versatility of their hydrogel.^69,70^ While PVA-based BE hydrogels exhibit many attractive properties, PVA is poorly soluble in water, requiring high temperature and/or alkaline conditions for dissolution, often making it difficult to use.^72,73^

Borax, which is a sodium borate salt, is another readily available molecule that has been investigated for BE cross-linking to form BE hydrogels.^74−76^ In fact, common polysaccharides were reported to undergo gelation when combined with borax in pioneering work in the field from the 1940s.^77^ Later, Leibler and colleagues investigated the molecular structure, phase behavior, and rheological properties of polysaccharide solutions cross-linked with borax.^78−80^ More recently, borax has been combined with PVA or dithiothreitol-modified PEG to form BE hydrogels at physiological pH. However, borax has known toxicity limiting its use in biomedical applications.^81,82^ Further, the use of a small molecule cross-linker in a dynamic system can lead to undesired molecule leakage and rapid hydrogel degradation as well as complex rheological behavior.

Overall, there are few molecules and polymers available for the straightforward design of BE hydrogels; and a careful chemical design of both PBA- and diol-modified polymers remains necessary to obtain BE hydrogels under physiological conditions. For effective BE cross-linking at physiological pH, the two aforementioned strategies—lowering the p*K*_a, acid_ of PBA and/or increasing the diol reactivity—have been investigated (Table 1). In the following sections, we elaborate on advanced methods to design and synthesize BE hydrogels under physiologic conditions for use in biomedicine.

**Table 1:**
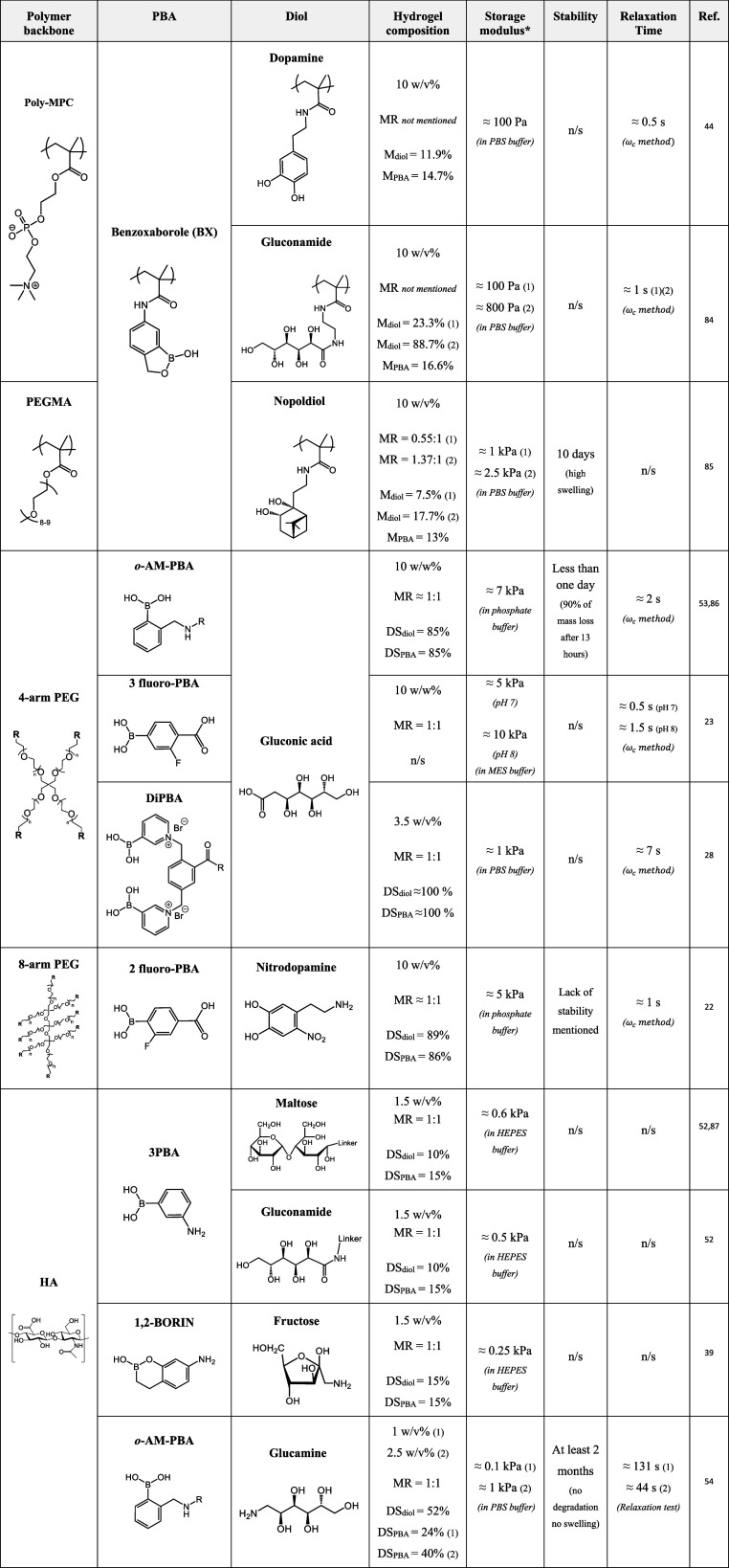
Boronate Ester Hydrogels Obtained at Physiological pH^a^

a MR: diol:PBA molar ratio, M: molar percentage in copolymer composition, DS: degree of substitution, *Storage modulus: *G*′_1 Hz_.

#### 3.1.2 Advanced Chemical Design of BE Hydrogels

As a PBA with a low p*K*_a, acid_, BX has been used in several studies for the design of BE hydrogels.^44,83,84^ For example, a vinyl copolymer bearing BX moieties formed hydrogels when combined with the same polymer bearing gluconamide or dopamine groups at pH 7.4.^44,84^ The 10 wt % hydrogels formed within 15 s and had *G*′_1 Hz_ values ranging from ≈100–800 Pa depending on the formulation. BE hydrogels at physiological pH were also obtained from BX-modified HA when combined with HA modified with gluconamide, fructose, or maltose, with degrees of substitution in the range of 10–15% and at a final polymer concentration of 1.5% (w/v).^52^ The combination of BX and fructose as a cross-linking pair led to similar rheological properties to PBA/fructose cross-linking, with a *G*′_1 Hz_ ≈ 200 Pa. The same authors investigated two six-membered ring homologues of BX: one with the boron atom connected to the phenyl ring (2,1-BORIN, p*K*_a, acid_ = 8.4), and the other one with the oxygen atom between the phenyl ring and the boron atom (1,2-BORIN, p*K*_a, acid_ = 6.17).^39^ Each of the two molecules was grafted onto HA before mixing with HA-fructose, with degrees of substitution in the range of 15% and at a final polymer concentration of 1.5% (w/v). The rheological properties of the obtained hydrogels were compared to those of hydrogels obtained via BX/fructose cross-linking. While 2,1-BORIN/fructose cross-linking led to limited gelation (*G*′_1 Hz_ ≈ 20 Pa), which was justified by the lack of ring strain in benzoxaborins, 1,2-BORIN/fructose cross-linking produced hydrogels with a *G*′ modulus higher than that of BX/fructose hydrogels (*G*′_1 Hz_ ≈ 250 Pa and ≈150 Pa, respectively). The authors justified this result by a strong electron-withdrawing effect of the oxygen atom in 1,2-BORIN that counterbalanced the lack of ring strain. These results are coherent with the decreasing order of calculated p*K*_a, acid_ for 2,1-BORIN, BX, and 1,2-BORIN (p*K*_a, acid_ of 8.4, 7.2, and 6.4, respectively), supporting the overall paradigm that designing PBAs with lower p*K*_a, acid_ leads to more effective BE cross-linking at physiological pH.

The unusually low p*K*_a, acid_ of *o*-AM-PBA (≈5.2) also makes it a potential candidate for BE cross-linking. Recently, we showed that HA-based hydrogels formed by specifically using *o*-AM-PBA and glucamine as a cross-linking pair.^54^ In this study, we first modified HA with a series of PBAs (i.e., 2-amino-PBA, 4-amino-PBA, BX, and *o*-AM-PBA) and diols (i.e., glucosamine, galactosamine, fructosamine, dulcitolamine, iditolamine, and glucamine). HA-PBAs and HA-diols were then systematically mixed to screen each potential binding pair in an equimolar PBA:diol ratio at 1% (w/v). The study revealed the unique property of the *o*-AM-PBA/glucamine pair to form hydrogels at pH 7.4, with a storage modulus (*G*′_1 Hz_ ≈ 175 Pa) substantially higher than that of any other combination. After optimization, we showed that these hydrogels are nonswelling and stable long-term in culture medium (at least 2 months in DMEM), which is relatively unique and critical for cell culture applications. We further showed that stable BE hydrogels can be designed over a range of viscoelastic properties (*G*′_1 Hz_ = 100–1000 Pa) and are cytocompatible, making them interesting candidates for biological investigations. Interestingly, others reported the use of *o*-AM-PBA/gluconic acid cross-linking in an equimolar ratio to form 4-arm PEG hydrogels with higher storage modulus (*G*′_1 Hz_ = 7 kPa).^53^ Taken together these two studies confirmed that the common sequence of asymmetric carbons shared by gluconic acid and glucamine has a specific affinity for *o*-AM-PBA, which allows for effective cross-linking in physiological conditions.

Taking advantage of the EWG effect of the fluorine group, BE hydrogels were also designed using fluoro-PBA, to enable cross-linking with diols at physiological pH. For example, fluoro-PBA/gluconic acid cross-linking was investigated using 4-arm PEG (10% [w/w]). This strategy allowed to produce BE hydrogels with a relatively high storage modulus (*G*′_1 Hz_ ≈ 5 kPa) and a short relaxation time (*τ*_*r*_ ≈ 1 s).^23^ Fluoro-PBA was also combined with nitrodopamine, which is a catechol derivative.^22^ This cross-linking pair formed an 8-arm PEG hydrogel (10% [w/v]) with a storage modulus (*G*′_1 Hz_ ≈ 5 kPa) and relaxation time (*τ*_*r*_ ≈ 1 s) similar to those of fluoro-PBA/gluconic acid cross-linking. While catechol derivatives are sensitive to oxidation, the authors mentioned that the presence of a nitro compound on the dopamine rendered the molecule less prone to degradation. Unfortunately, the study did not provide the swelling/stability profile of the obtained hydrogels and only mentioned a lack of stability in physiological conditions, making it difficult to conclude on the potential use of this cross-linking strategy.

Regarding diol design, the most significant breakthrough is related to the use of nopoldiol, a synthetic bicyclic diol. Chen et al. developed BE hydrogels from the combination of two PEGylated vinyl copolymers containing either a methacrylated BX or a methacrylated nopoldiol monomer. Mixing the two polymer solutions led to instantaneous gelation and produced hydrogels with *G*′_1 Hz_ ≈ 1 kPa, which indirectly confirmed efficient BE formation between nopoldiol and BX.^85^ Interestingly, the BX/nopoldiol hydrogels did not degrade in the presence of competitive sugars (i.e., d-glucose and fructose) over the course of a 2-h experiment. Conversely, degradation was observed for BX/gluconic acid hydrogels, which suggests that nopoldiol has a higher binding capacity toward BX than gluconic acid. However, BX/nopoldiol hydrogels swelled up to twice their initial mass and collapsed within 10 days of immersion at pH 7.4. The authors further demonstrated that complete hydrogel degradation can be delayed by 10 days when introducing gluconic acid moieties within the nopoldiol-bearing vinyl polymer for BE cocrosslinking. The authors hypothesized that the different degradation profiles resulted from a difference in their cross-linking densities. One can also hypothesize that the observed differences are linked to the distinct esterification/hydrolysis kinetics of each PBA/diol.

### 3.2 Physicochemical Properties of BE Hydrogels and Limitations

The dynamic covalent nature of BE bonds provides hydrogels with a variety of interesting physicochemical properties, such as self-healing, injectability, and viscoelasticity. Viscoelasticity is commonly characterized by the storage modulus *G*′ and loss modulus *G*′’ of the hydrogels. It is also associated with a relaxation time, i.e., the time for network reorganization and maximal energy dissipation upon strain. Several studies have demonstrated that BE hydrogels relax stress rapidly, with complete relaxation within seconds to tens of seconds.^22,28,54^ Relaxation properties of BE hydrogels are only sparsely reported. More importantly, the variety of polymer backbones and cross-linking pairs reported makes the direct comparison of the relaxation behaviors of the existing systems difficult. Typically, the use of polysaccharides (e.g., HA, alginate) with distinct DS, molecular weights, and degrees of entanglement, add to the complexity of the physical characterization and understanding of BE hydrogels. Yet, a few systems focused on the use of 4-arm PEG to design what could be considered ideal polymer networks,^88^ allowing us to draw some conclusions. These systems all used gluconolactone as a diol as well as similar formulations (i.e., DS, molar ratio, polymer content) but distinct PBA derivatives (i.e., PBA, fluoro-PBA, and *o*-AM-PBA).^23,86,89^ Put into perspective, these studies reveal an increase in relaxation time in the following order: PBA < fluoro-PBA < *o*-AM-PBA. This order is consistent with the higher affinity of fluoro-PBA and *o*-AM-PBA toward diols. In general, the fact that the relaxation time of common BE hydrogels does not exceed tens of seconds defines them as fast-relaxing networks. Fast relaxation enables BE hydrogels to flow and be deformed without losing their integrity and mechanical properties, making them particularly interesting as injectable and malleable materials. However, fast relaxation is usually associated with relatively low elastic properties, with *G*′ values in the range of hundreds to a few thousands of pascals. Other classes of dynamic covalent bonds such as Schiff bases (e.g., imine, hydrazone, oxime) allow hydrogel relaxation times from seconds to hours.^90,91^ In this context, more work needs to be done to expand the relaxation properties of BE hydrogels.

Hydrogel stability under physiological conditions is an essential feature for most biomedical applications. This implies minimal swelling/shrinking after immersion in buffer solution or culture medium, as well as the lack of degradation over weeks to months. However, the reversible nature of BE bonds, in combination with low binding constants, make BE hydrogels sensitive to osmotic forces and prone to degradation. Thus, the few stability studies reported to date revealed that BE hydrogels tend to degrade in physiological media within hours to days.^53,85^ Additionally, BE hydrogels stability can be highly affected by ROS such as H_2_O_2_, which can react with PBA and lead to deboronation.^92,93^ In the deboronation process, an oxygen atom first binds to the boron atom on PBA prior to a molecular rearrangement consisting of the insertion of the oxygen atom between the boron and the aromatic ring. It is followed by the hydrolysis of the B–O bond to form boric acid and phenol. Of note, boric acid and phenol are toxic,^65,66^ which is a point of vigilance for the use of BE hydrogel in highly ROS-concentrated environments. In this context, we recently reported a new class of BE hydrogels that are stable for at least two months in phosphate buffer and culture medium. These were obtained by mixing two HA components modified with *o*-AM-PBA and glucamine, in a PBA:diol molar ratio of 1:1.^54^ To date, most reported BE hydrogels used the end-functionalized 4- or 8-arm PEG as polymer backbones.^23,53,94^ In these systems, the limited number of reactive functional groups per polymer chain, in combination with the low binding constants of BE precursors, may limit the ability of each polymer chain to remain connected to the polymer network. Thus, we hypothesized that the improved stability of our hydrogels was the result of a high number of reactive groups per HA chain. This allows higher cross-linking density for a given polymer content as compared with telechelic polymers, such as PEG, and may contribute to better anchoring polymer chains within polymer networks. To circumvent the issue of limited stability, research has been dedicated to combining BE cross-linking with covalent cross-linking.^22,95^ For example, Tang et al. developed a fast-relaxing (≈2 s) and relatively stiff (*G*′_1 Hz_ = 5 kPa) PEG-based hydrogel by combining fluoro-PBA/nitrodopamine cross-linking with strain-promoted azide–alkyne cycloaddition (SPAAC) covalent cross-linking.^22^ The authors showed that only BE hydrogels with additional covalent cross-linking were stable in culture conditions. While they claimed that the materials maintained rapid stress relaxation with their co-cross-linked system, this approach should be carefully considered because incorporating covalent bonds within dynamic hydrogels can affect their viscoelastic properties.^96^ It also makes hydrogel design more complex, and thus less translatable. To conclude on stability, one should keep in mind that BEs are not bioorthogonal due to the presence of natural polyols in biological systems, and that BE dynamic equilibria and properties are easily affected by the buffer or medium composition.^93^ In particular, using the PBA/ARS pair in water, early works investigated the effect of salts on BE formation and stability and showed that changing the phosphate concentration affects equilibrium constants.^20,97^ A recent study further confirmed the strong and positive effect of phosphate salts on BE formation in a mixed solvent (acetone/water).^98^ Beyond the salt composition, it is known that PBA/diol interactions differ in organic and aqueous conditions.^99^ In this regard, the reported values of equilibrium constants determined in organic solvents are often difficult to translate to applications in physiological conditions, and many systems require further evaluation in relevant solvent/buffer conditions. Overall, evaluating the effect of medium on the viscoelasticity and stability of new BE hydrogels is a key point in their development and translation.

Strain stiffening is a property that is less explored for BE hydrogels. Strain stiffening describes an increase in material stiffness upon deformation, which is frequently observed in biological tissues.^100^ This property was recently reported for the first time for a BE hydrogel.^94^ Using a combination of fluoro-PBA-modifed 4-arm-PEG and gluconic acid-modified 4-arm-PEG, the authors showed that the intensity of strain stiffening was inversely proportional to both network elasticity (i.e., storage modulus, *G*′) and critical stress, which is the maximum stress before transitioning from a viscoelastic to a strain-stiffening dominant behavior. They demonstrated that both lowering pH from 8 to 6.5 and increasing temperature from 5 to 35 °C favored strain stiffening and lowered the critical stress. This behavior may be generalizable to all BE hydrogels and will deserve more attention in the future, possibly opening the way for new biomimetic hydrogel design.

Overall, despite significant progress in the chemical design of PBA/diol cross-linking schemes, the low binding constants associated with BE formation commonly produce hydrogels that are weak (i.e., low storage moduli), have a limited range of viscoelastic properties and relaxation times, and are rarely stable in culture conditions. These observations highlight the need for further investigating BE cross-linking strategies, which can benefit from the development of innovative PBA derivatives reported in other fields of research.

### 3.3 New Perspectives in Boronate Ester Hydrogel Design

To improve BE hydrogel stability, one option is the development of new PBA/diol pairs with higher binding capacity. Iminoboronate esters developed in other contexts can serve as a source of inspiration (Table S1). Iminoboronate esters describe BE functionalities stabilized by an adjacent imine group, which is obtained via Schiff base formation between a carbonyl and a primary amine.

To obtain an iminoboronate ester, a first strategy consists in forming a three-component assembly upon reaction of an *ortho*-carbonyl-PBA, a primary amine, and a diol.^101^ Here, the imine stability can be adjusted depending on the nature of the carbonyl group (i.e., aldehyde or ketone) and that of the primary amine. Typically, the reaction of 2-formyl-PBA with glycine, 2-methoxyethylamine, acethydrazide, benzhydrazide, or 6-aminoxyhexanoic acid have equilibrium constants (K_eq_)^1^ of 16 M^–1^, 81 M^–1^, 1,666 M^–1^, 2,000 M^–1^, and 71,428 M^–1^, respectively.^102,103^ As an alternative to an adjacent Schiff base, a thiazolidine group, which is hardly reversible in physiological conditions,^104^ can be obtained by reacting cysteamine and 2-formyl-PBA. One study demonstrated that the three-component assembly strategy produces iminoboronate ester with much stronger binding than BE formation alone.^105^ They showed that the addition of benzylamine as a third component to the reaction between catechol and 2-formyl-PBA increased the binding constant from 112 M^–1^ to 2.45 × 10^3^ M^–1^. The three-component assembly was once used for the design of a complex guanosine-based supramolecular hydrogel.^106^ In this system, 2-formyl-PBA reacted on one side with the multiple primary amines of aminoglycosides via Schiff base formation and, on the other side, with the diol groups of guanosines on a guanosine-based supramolecular assembly via BE formation. The authors showed that the obtained iminoboronate ester-based supramolecular hydrogel had interesting antibacterial activity in addition to pH and glucose sensitivity; however, they did not investigate the effect of iminoboronate ester bonds as compared with BE bonds on the rheological properties of the hydrogel.

A second strategy consists in combining an *ortho*-carbonyl-PBA with an aminated diol. While less studied, this two-component approach was notably developed for in vivo targeting, where NHS-modified 2-formyl-PBA was intradermally injected to react with amines in the extracellular matrix prior to a systemic injection of a fluorescent nopoldiol derivatives containing an adjacent primary amine.^107^ The authors observed the persistence of the fluorescent signal at the intradermal site of injection even after 7 days, suggesting successful targeting and high stability of the obtained iminoboronate esters, which they described as practically irreversible. This system could easily be transposed to hydrogel cross-linking. While irreversible cross-linking may not be desirable, the nature of the Schiff base could then be easily adjusted (i.e., imine, hydrazone, or oxime) to tune the adduct stability, allowing for physicochemical adjustments.

Taking advantage of the long history of BE chemistry, new alternatives to diols could also be investigated (Table S1). In particular, it is known that salicylic acid derivatives, especially salicylhydroxamic acid (SHA), bind strongly to BA.^108−110^ For example, SHA was used in sepharose purification columns to purify PBA-modified proteins.^111,112^ Based on ^11^B NMR analysis, the authors suggested that PBA/SHA binding could occur either through BE formation (i.e., 5-membered ring structure) or the formation of a 6-membered ring involving a hydroxyl group and the amine of SHA. A more recent study reported the binding constants of several PBA/SHA derivative pairs.^113^ Among the different SHA derivatives tested (i.e., SHA, *N*-methyl-SHA, *ortho*-hydroxyl-SHA, and 2,5-dihydroxy-1,4-benzdihydroxamic acid), SHA and *ortho*-hydroxyl-SHA had the highest K_eq_ values at physiological pH (1.6 × 10^4^ and 3.6 × 10^4^ M^–1^, respectively), making them interesting candidates for BE cross-linking. To date, a single hydrogel system based on PBA/SHA cross-linking has been reported.^114,115^ The authors presented the successful synthesis of viscoelastic hydrogels from PBA- and SHA-modified vinyl polymers, with a *G*′_1 Hz_ of ≈500 Pa and *τ*_*r*_ ≈ 20 s at pH 7.6. The authors did not report on the hydrogel stability, which does not allow us to conclude on the potential advantage of using SHA derivatives over other diols. To our knowledge, this cross-linking pair reported ten years ago was not further investigated, which calls for more studies in this direction.

Beyond improving stability, advanced chemical design could provide BE hydrogels with new properties. For example, bisboronic acids could be investigated for enhanced sugar sensitivity and detection. Bisboronic acids are molecules that contain two adjacent BAs with a well-known high affinity toward some compounds with multiple diols, especially glucose.^116,117^ To date, a single BE hydrogel based on the use of a bisboronic acid was reported.^28^ The authors first demonstrated that a molecule containing two adjacent PyBA, which they called DiPBA, can react with gluconic acid to produce a 4-arm PEG hydrogel at physiological pH, with a *G*′_1 Hz_ of 170 Pa and fast relaxation (*τ*_*r*_ ≈ 7 s). The authors then confirmed the increased glucose binding capacity of DiPBA compared to PyBA and fluoro-PBA, with K_eq_ of 1295 M^–1^, 164.7 M^–1^ and 8.6 M^–1^, respectively. On the contrary, no difference in gluconic acid binding capacity was observed, which is consistent with the hypothesis of a single binding site for gluconic acid. Then, they demonstrated that the addition of glucose to the buffer (PBS) prevented hydrogel formation whereas the addition of fructose or lactate had no impact on the final rheological properties of the hydrogel (*G*′ = 166 and 140 Pa, respectively) compared with gels in PBS alone. As a proof of concept, they further demonstrated that the profile of insulin release from their DiPBA/gluconic acid hydrogel depended on glucose concentration while it was not affected by lactate. This work is a nice example of how advanced BE hydrogel design enabled glucose responsiveness with high specificity, which is more relevant for clinical translation.

Following a different path, Accardo et al. developed an original BE hydrogel with photocontrolled gelation.^118,119^ To succeed, the authors used a PBA modified with an azobenzene group, whose E/Z isomerization can be controlled with light. It was previously demonstrated that the E/Z isomerization of an azobenzene group on the *ortho* position of catecholborane affects the catecholborane Lewis acidity.^120^ In this study, the authors hypothesized that the E/Z isomerization of an *ortho*-azobenzene-PBA could influence the BE equilibrium as well, and thus influence the BE hydrogel properties. They demonstrated a 4.3-fold increase in K_eq_ for the Z-isomer compared to the E-isomer when mixed with a model diol molecule (i.e., pinacol). Then, they showed that a 4-arm-PEG modified with *ortho*-azobenzene-PBA in combination with a gluconic acid-modified 4-arm-PEG formed a hydrogel under physiological conditions only when switching the azobenzene group from E to Z isomer with UV light (365 nm). They demonstrated that the elastic properties of the hydrogel could be adjusted with light exposure time. Typically, over 2 h of UV light exposure, they observed a progressive increase in *G*′_1 Hz_ from 0 to 170 Pa. They further showed that reversing isomerization under blue light (470 nm) reversed gelation within minutes. Interestingly, by substituting the *ortho*-azobenzene with a difluoro-azobenzene group that is sensitive to green and blue lights, they achieved on/off gelation under 525/470 nm light exposure, constituting the first BE hydrogel with on-demand gelation and dissolution entirely under visible light. This promising approach is, however, mostly based on hour-long exposure times, which is limiting and calls for further optimization.

## 4 Biomedical Applications

PBAs with strong and/or specific interaction with diols were first developed to improve purification and analytical techniques. They were notably used in purification columns with the aim to isolate biomolecules, including monosaccharides, nucleosides, and glycoproteins.^121−124^ These innovations inspired new technological developments for the sensing and release of small biological molecules, with application in drug delivery.^43,125−127^ The more recent development of BE hydrogels has open a new era of research, with applications in local drug/cell delivery, 3D cell culture, and biofabrication.

### 4.1 Drug Delivery Systems

Hydrogels, especially injectable hydrogels, are instrumental in the design of local drug delivery systems. Injectable hydrogels enable straightforward drug encapsulation and delivery to a target site, limit the fast clearance of drug by the body fluids, and can be tuned to avoid burst release.^128−131^ The physicochemical properties of an injectable hydrogel play an important role in the passive drug release kinetics. As such, the composition of a hydrogel developed for drug delivery should be carefully considered, including attention to the nature of the polymer and reactive functional groups, the polymer content, and the cross-linking density.

BE hydrogels, which are dynamic covalent networks, are injectable owing to the reversible nature of the cross-links, and thus enable minimally invasive delivery.^132^ BE hydrogels can be used as carriers for local drug delivery, where loaded drugs are passively released by either spontaneous diffusion or hydrogel degradation.^53,133^ More importantly, BEs are highly sensitive to their biochemical environment and they can respond to a variety of stimuli, including changes in pH, the presence of ROS, and the presence of glucose, making them attractive functional groups for local triggered drug release.^134^

#### 4.1.1 Glucose-Responsive BE Hydrogels

BE hydrogels are commonly used as glucose-responsive hydrogels. Free glucose can react with PBAs and compete with diols within a BE hydrogel network. This can alter the hydrogel mesh size and sometimes its integrity, which in turn can trigger drug release. For this reason, BE hydrogels have been developed for innovative treatments for diabetes.^135−140^ Typically, BE hydrogels have been designed for on-demand insulin delivery, where insulin delivered in a BE hydrogel can be released specifically during episodes of hyperglycemia (blood glucose >10 mM).^141^ A study reported that 35% of the insulin encapsulated in a BE hydrogel was released after 1 day of immersion in high glucose medium (≈ 17 mM) while only 7% was released over the same period of time in PBS.^142^ In a different study, a microneedle patch, which is convenient for transdermal application,^143^ was developed for insulin delivery using a BE hydrogel.^144^ To form their hydrogel, the authors combined a synthetic copolymer containing PyBA moieties and gluconic acid-modified 4-arm-PEG. The hydrogel was loaded with insulin before being molded into a microneedle patch. The authors demonstrated an increase in insulin release from 45% to 80% after 9 h when adding glucose (22 mM) to PBS. They also reported an efficient reduction (from 22 mM to ≈10 mM) in blood glucose level (BGL) in a diabetic rat model equipped for 4 h with the insulin patch, which was not observed with the unloaded patch control (BGL = 26 mM). Of note, the activity of glucose oxidase, which catalyzes the oxidation of glucose into gluconic acid, has also been highlighted as a process that can potentially alter BE hydrogels in vivo.^145^ Following an increase in glucose concentration, this enzymatic reaction can locally increase the amount of gluconic acid, which is known to have a relatively high affinity toward PBA. This reaction can also lower the pH and, thus, destabilize BE networks based on their pH sensitivity.^146^

#### 4.1.2 ROS- and pH-Responsive BE Hydrogels

**Figure 2:**
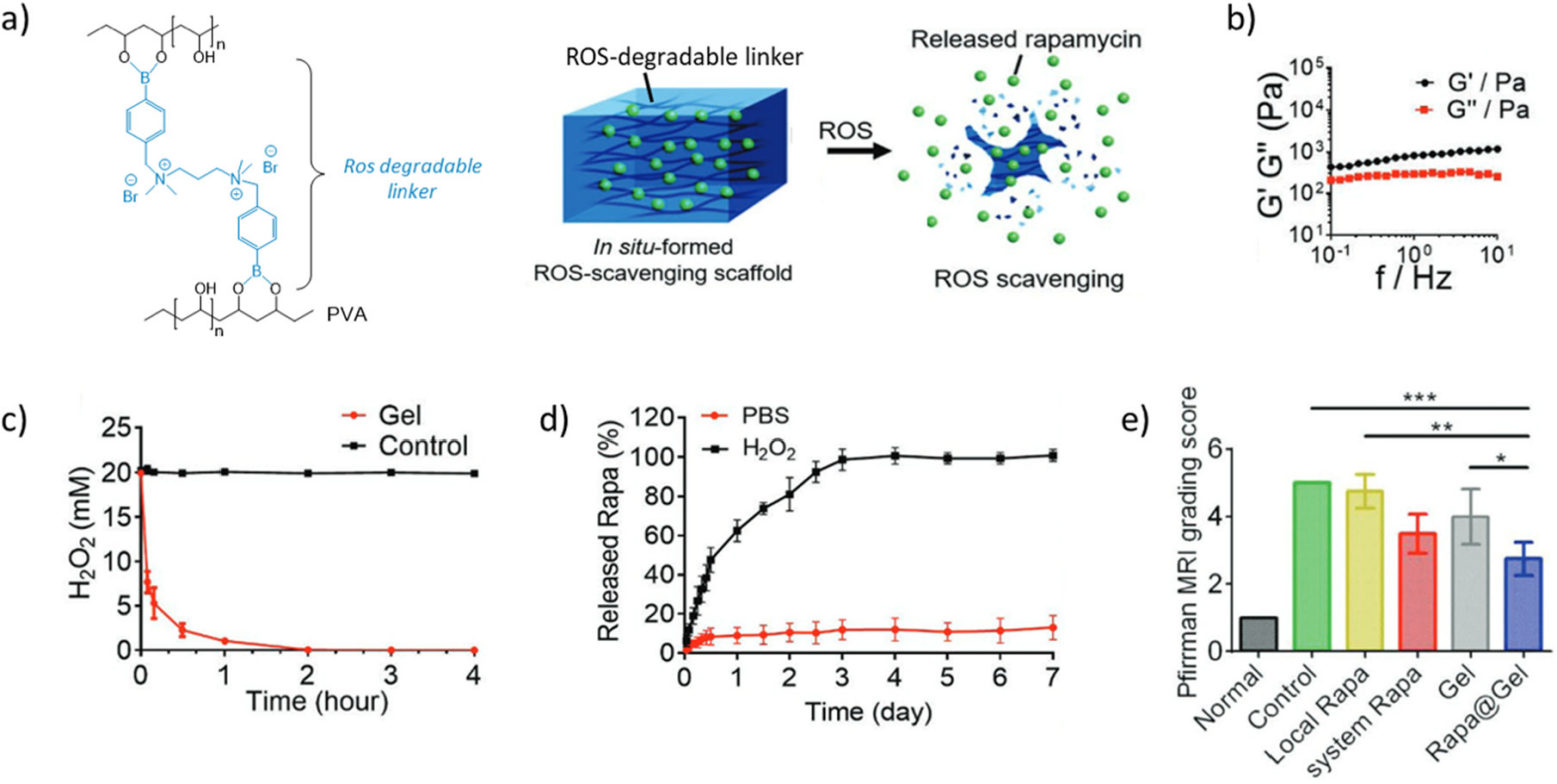
Rapamycin delivery from a boronate ester hydrogel for the treatment of the intervertebral disk degeneration. (a) TSPBA/PVA hydrogel formulation and schematic of rapamycin-loaded ROS-responsive hydrogel. (b) Storage and loss moduli of the hydrogel loaded with rapamycin. (c) Addition of TSPBA/PVA hydrogel to H_2_O_2_ (20 × 10^–3^ M), and evaluation of H_2_O_2_ content by titanyl sulfate shows the ROS-scavenging effect of the TSPBA/PVA hydrogel formulation. (d) Cumulative release of rapamycin from hydrogels in PBS limited to less than 20% without H_2_O_2_ and complete after 4 days with H_2_O_2_ (1 × 10^–3^ M). (e) Significantly reduced Pfirman MRI score using the hydrogel-encapsulated rapamycin compared to the local rapamycin delivery, confirming the therapeutic effect in a rat model. (adapted with permission from Bai et al.^151^). Copyright [2023][John Wiley & Sons].

ROS and pH are also known to affect the physicochemical properties of BE hydrogels. Thus, variations in ROS levels and pH have also been investigated as stimuli for on-demand drug release from BE hydrogels. Interestingly, ROS-induced deboronation makes BE hydrogels potential ROS-scavenging materials that can protect cells from oxidative stress, inflammatory microenvironment, and tumor development.^147−150^ One study reported the potential use of ROS-sensitive BE hydrogels for the treatment of intervertebral disk (IVD) degeneration (Figure 2).^151^ In this study, the authors validated that a BE hydrogel composed of a di-PBA cross-linker and PVA showed a ROS scavenging capacity in a high concentrated H_2_O_2_ (ROS) medium that mimics the degenerated IVD environment. As the ROS-induced deboronation decreases the amount of reactive PBA within the network, then destabilizes it, they used this BE hydrogel as a ROS-sensitive delivery system of rapamycin, which is an immunosuppressive drug. They validated that rapamycin encapsulation did not alter the rheological properties (*G*′_1 Hz_ ≈ 1 kPa) of the hydrogel. When immersing the hydrogel in PBS, the addition of H_2_O_2_ (1 × 10^–3^ M) to PBS induced a release of nearly 100% of the loaded rapamycin after 3 days, whereas less than 20% was released in PBS alone. Compared with rapamycin alone in an IVD degeneration rat model, the rapamycin-loaded hydrogel led to an improved IVD condition as evaluated by MRI, as well as an increase in the disc height index.^152^ These results led to a significant decrease in IVD degeneration index (i.e., MRI Pfirrmann score of ≈2.5 vs 4.5 for the rapamycin-loaded hydrogel and the rapamycin alone conditions, respectively). Surprisingly, the in vivo results obtained for the hydrogel alone and rapamycin alone conditions did not show significant therapeutic effects compared to the untreated control group (without injection). Thus, the positive results of the rapamycin-loaded hydrogel could suggest a synergy between rapamycin and the hydrogel formulation. Although not applied to BE hydrogels, recent studies reported an inspiring use of deboronation to achieve ROS-responsive molecular release.^153,154^ The approach is based on the fact that a phenol ester-linked molecule can undergo a 1,6-elimination reaction that cleaves off a quinone-methide molecule to release the original carboxylic acid-functionalized molecule. Upon exposure to ROS and following deboronation, a PBA ester-linked molecule is converted to a phenol ester-linked molecule that can then rearrange and free the molecule of interest. Initially developed for peroxide detection, the so-called self-immolative boronic acid linker has been used for the delivery of an antithrombotic drug (i.e., all-trans retinoic acid) from nanoparticles in response to ROS.^155^ It holds great potential for the design of ROS-responsive drug delivery systems based on BE hydrogels.

Regarding cancer treatment, the abnormal ROS level and acidic pH (≈ 6.5) of the tumor microenvironment can be exploited for tumor-responsive drug delivery from BE hydrogels. For example, a study reported the use of a BE hydrogel composed of HA-dopamine cross-linked with PBA-modified nanoparticles for the local delivery of an anticancer drug (doxorubicin, DOX).^156^ The authors demonstrated that acidic pH (6.5) accelerated hydrogel degradation compared to neutral pH, with a remaining mass of ≈40% and ≈80%, respectively, after 8 days of immersion. The degradation was accompanied by a DOX release of 40% at acidic pH after 8 days as compared with less than 10% release at neutral pH. Using an incomplete tumor resection model in mice, the authors also showed limited tumor growth after injection of the DOX-loaded hydrogel (≈ 250%) compared to the hydrogel alone (≈1,800%); however, the results were not significantly different from DOX alone (≈500%) due to high variability in the results. Finally, the DOX-loaded hydrogel treatment also prevented some side effects from the anticancer drug treatment, with an improved mice body mass of 110% of the initial mass compared to an abnormal 15% loss of weight for the DOX alone treated group at 20 days.

#### 4.1.3 Boronate Ester-Based Sustained Drug Release

To sustain drug release from a BE hydrogel, another strategy takes advantage of diols naturally present on a therapeutic molecule. In this approach, the delivered drug can form reversible covalent bonds with the hydrogel network, which slows its diffusion out of the hydrogel scaffold. For example, De Oliveira et al. studied the controlled delivery of dihydrocaffeic acid (DHCA), an antiphotoaging agent, from a BE hydrogel composed of HA-gluconic acid and HA-PBA, for UV skin protection.^157^ In this system, the catechol moieties of encapsulated DHCA were expected to reversibly bind to PBA groups, which should lead to sustained DHCA release. Using an excess of PBA to gluconic acid (molar ratio of 2:1), the authors showed that DHCA encapsulation did not affect the storage moduli of the BE hydrogel with a *G*′_1 Hz_ of 296 and 305 Pa for the hydrogel with and without DHCA, respectively. Slow DHCA release with no burst was observed, with only 9% of DHCA released after 8 h at pH 7.4. However, since DHCA naturally carries the catechol moiety, no control group without PBA binding could be tested. The authors also showed that the reduced binding constants of PBA/DHCA at acidic pH (K_eq_ of 1,727 M^–1^ and 131 M^–1^ at pH 7.4 and 6, respectively) led to accelerated release under acidic conditions, with 19% of released drug at pH 6 after 8 h, which is more representative of the treatment conditions as skin pH is acidic. Compared to the nontreated condition in vitro, they showed that only a high concentration (35 mM) of DHCA alone protected fibroblasts from UVB irradiation (600 mJ/cm^2^, 24 h), whereas a low dose of DHCA (7 mM) was enough to significantly improve cell viability when encapsulated in the BE hydrogel. This in vitro study did not allow the authors to conclude on the benefits of a sustained release strategy, motivating the need for future in vivo investigations.

Polyphenols, which have antibacterial and antioxidative properties, commonly comprise several catechol groups. Thus, when delivering a polyphenol as a drug, the drug itself can be used as a cross-linker for BE hydrogel design. In other words, the delivered polyphenol can become a part of the delivery scaffold and play a role in its own sustained release. For example, ellagic acid (EA), which is a natural polyphenol found in fruits, successfully formed hydrogels when mixed with nitroPBA-modified 8-arm-PEG.^21^ With this system, 50% of EA was released in 5 days followed by a steady but slow release up to 62% over 15 days. A similar approach was used with epigallocatechin-3-gallate (EGCG), another polyphenol, delivered from a chitosan-based BE hydrogel for wound healing.^45^ Interestingly, this study reported a release profile similar to the previous study, yet over a shorter time period, with 35% of EGCG released in 2 days, followed by a steady but slower release up to 45% over 4 days under acidic condition (pH = 5).

To sustain the release of a protein drug from a BE hydrogel, Ali et al. developed a different strategy where PVA and a protein of interest were reversibly cross-linked with a heterobifunctional linker.^158^ In this system, the linker contained both a PBA to form BE with PVA and an aldehyde to form imines with protein lysines. Using bovine serum albumin (BSA) as a model protein, they showed that, even if the protein was part of the hydrogel network, the reversibility of both BE and imine bonds ensured a protein release of around 30% after 3 h under physiological conditions, followed by a release plateau. Of note, the use of a second type of reversible bond makes the hydrogel design more complex, and the proposed linker itself can diffuse out of the hydrogel and interact with surrounding tissues. Thus, a more direct chemical design where the molecule of interest is modified with PBA or diol moieties may be more desirable. As early as 1994, Shiino et al. made the proof of concept of protein (insulin) modification with gluconic acid molecules for glucose-mediated insulin release from PBA-modified beads.^135,159^ They demonstrated that the release of the gluconic acid-modified insulin depended on glucose concentration, with an increase in glucose concentration resulting in an increase in insulin release. However, unmodified insulin was not used as a control, which does not allow proper assessment of the utility of the chemical modification. This bead-based strategy could be transposed to BE hydrogels for the development of a new generation of local drug release systems.

### 4.2 Boronate Ester Hydrogels for 3D Cell Culture

Over the last 20 years, 3D systems have been increasingly used to culture cells in vitro. 3D cell culture systems provide cells with a more biologically relevant microenvironment, where cell–cell and cell–extracellular matrix (ECM) interactions can be better reproduced.^160^ These systems are now instrumental in producing cells and organoids for healthy and diseased tissue modeling and the development of new therapies.^161,162^ In this context, hydrogels have attracted increasing attention because they can mimic the hydration, polymer composition, and physical properties of the natural ECM.^6^ Among the physical properties of the ECM, its viscoelasticity has recently been identified as a key parameter to guide cell functions, including spreading,^163,164^ proliferation,^165^ and differentiation.^166^ Yet, much remains unknown regarding the effect of viscoelasticity on cell behavior, calling for the development of new viscoelastic hydrogels for 3D cell culture. While covalently cross-linked hydrogels lack viscoelasticity, it is an inherent property of noncovalent and dynamic covalent hydrogels.^167^ This makes BE hydrogels particularly interesting for biological investigations.^39,44,85^

#### 4.2.1 From Cell–Matrix Interactions to Cell Fate

To study cell–matrix interactions in soft tissues, Tang et al. developed a fast-relaxing hydrogel using BE cross-linking.^22^ Using fluoro-PBA/nitrodopamine cross-linking, they designed a PEG-based hydrogel with a *G*′_1 Hz_ ≈ 10 kPa and a relaxation time of ≈2 s. The system was further co-crosslinked via SPAAC to ensure stability under culture conditions, which, according to the authors, did not affect the relaxation time of the hydrogel. This system was used to encapsulate bone marrow mesenchymal stem/stromal cells (BMSCs) with a satisfactory viability of >90% after 7 days. BMSCs encapsulated in fast-relaxing hydrogels had a higher volume and lower sphericity than those encapsulated in purely elastic (SPAAC) hydrogels. Further investigations on the transcriptional coactivators YAP and TAZ, which influence cell mechanosensing, highlighted a change in subcellular YAP/TAZ localization from the cytoplasm to the nucleus. These results are consistent with previous results describing increased cell spreading in viscoelastic hydrogels.^163,164^ In a different study, Liu et al. investigated the effect of viscoelasticity on MSC spreading and differentiation, using a series of gelatin-based hydrogels differing in their rheological properties by the addition of BE bonds.^168^ To succeed, methacrylated gelatin (GelMA) was mixed with varied amounts of PBA-modified gelatin and 3,4-dihydroxybenzaldehyde, which is a small molecule cross-linker able to bind both gelatin (i.e., imine formation with primary amines) and PBA (i.e., boronate ester formation), before UV photo-cross-linking. Viscoelasticity was assessed via rheological measurements. Increasing PBA-modified gelatin concentration from 0% to 15% increased the *G*′′/*G*′ ratio (tan δ) from ≈0.05 to ≈0.5 while maintaining a constant *G*′_1 Hz_ of ≈200 Pa. The authors also successfully obtained a hydrogel with rheological properties matching those of bone marrow (i.e., tan δ ≈ 0.3 and *G*′_1 Hz_ ≈ 4 kPa). Seeding BMSCs on top of the hydrogels, the authors first showed that the ability of BMSCs to migrate in the hydrogels increased when increasing the tan δ. Interestingly, BMSCs did not migrate in purely covalent hydrogels (i.e., absence of PBA-modified gelatin) with similar *G*′_1 Hz_, suggesting an active role of viscoelasticity in their ability to migrate. After 7 days of culture in basal medium, BMSCs seeded on top of low viscoelasticity hydrogels (tan δ ≈ 0.2) vs elastic hydrogels (tan δ ≈ 0) showed a 2-fold and 5-fold increase in the gene expression of *Col2a1* and *Sox9*, respectively, which are two chondrogenic markers. Further increasing the tan δ to ≈0.3 led to 5-fold and 10-fold increases in *Col2a1* and *Sox9* expression, respectively. A similar increase in osteogenic markers, including *Alpl*, *Col1a1*, and *Runx2*, with increasing viscoelasticity was observed. Conversely, adipogenic markers (i.e., *Lpl*, *Plin1*, *Scd*) were downregulated. Interestingly, the authors also demonstrated an increase in the nuclear to cytoplasmic ratio of YAP, smad 2/3, and β-catenin with increased viscoelasticity. They hypothesized that viscoelasticity could activate YAP and promote smad2/3 and β-catenin nuclear translocation, which is consistent with the fact that chondrogenesis and osteogenesis are regulated by the TGF-β/SMAD and Wnt/β-catenin signaling pathways.^169−171^ Of note, this study was performed with cells seeded on top of the hydrogels, calling for similar investigations with encapsulated cells.

#### 4.2.2 BE Hydrogels As Coculture Scaffolds

**Figure 3:**
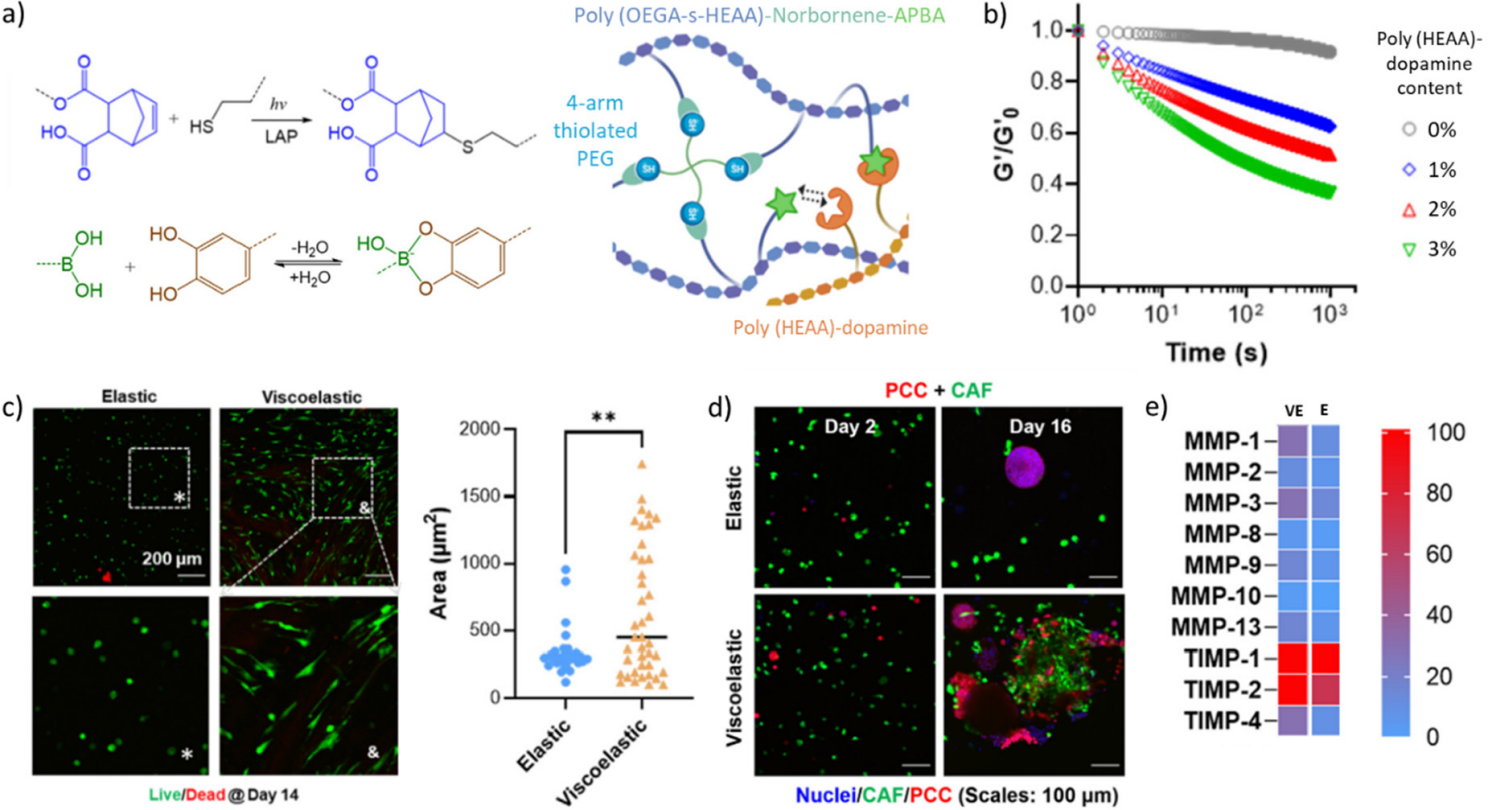
Comparison of elastic and viscoelastic boronate ester hydrogels for the study of pancreatic cancer cell phenotype, interactions, and secretion, alone and in co-cultured conditions. a) Light mediated thiol-norbornene and reversible boronate ester cross-linking reactions for the design of tunable viscoelastic hydrogels. (b) Effect of poly(HEAA)-dopamine content on stress-relaxation. An increase in dopamine content implies an increase in boronate ester formation, which induces an increase in stress relaxation. (c) Increase in stress relaxation of the hydrogel induces more spreading of CAFs visible through live/dead staining (days 14) with a loss of circularity and an increase in cell area compared to the elastic hydrogel scaffold. (d) Co-culture of encapsulated PCCs and CAFs in viscoelastic hydrogels induces closer interactions between the two cell types and an invasive phenotype for PCCs compared to the co-encapsulation in elastic hydrogels. (e) Co-encapsulated PCC/CAF secretome in viscoelastic and elastic hydrogels. Matrix metalloproteinase (MMPs) secretion increases in viscoelastic hydrogels, which is a consistent with the observed cell spreading and invasion (adapted with permission from Lin et al.^172^). Copyright [2023][Elsevier]

BE hydrogels were also used to evaluate the effects of matrix stress relaxation on cancer cell development (Figure 3).^172^ Here, the authors designed a poly((N-(2-Hydroxyethyl)acrylamide) copolymerized with norbornene by RAFT polymerization and mixed it with thiol-functionalized 4-armPEG and LAP for subsequent thiol–ene photo-cross-linking under UV irradiation (λ = 365 nm, 5 mW/cm^2^, 2 min). Viscoelasticity was introduced via the introduction of PBA moieties on one block copolymer and that of dopamine moieties on another block copolymer, which resulted in BE bonds forming in the polymer network. Upon addition of BE cross-linking, the viscous component (*G*′’) of the photo-cross-linked hydrogels increased significantly, with tan δ values of ≈0.025 and 0.2 for non-BE and BE hydrogels, respectively. While non-BE and BE hydrogels had similar *G*′ values (≈ 5 kPa), only BE hydrogels had stress relaxation properties, with a half-relaxation time (τ_1/2_) of 1,533 s. The authors showed that adding stress relaxation to the network significantly increased the spreading of cancer-associated fibroblasts (CAFs) and decreased cell circularity. They further took advantage of the cell spreading capacity in viscoelastic hydrogels to study CAF and pancreatic cancer cell (PCC) behavior in coculture conditions. In purely elastic hydrogels, PCCs grew in spheroids but separately from CAFs. Conversely, in viscoelastic hydrogels, the two cell types, initially nonhomogeneously distributed, were able to migrate together and form a well-mixed population. Furthermore, PCC formed irregular clusters with outgrowth, which is more representative of cancer cell proliferation according to the authors. The PCC invasive phenotype was then studied in the viscoelastic hydrogels. Compared to PCCs alone, PCCs in coculture with CAFs were characterized by an increase in the gene expression of matrix-degrading proteins (e.g., CTSK, matrix metalloproteinase [MMP]-1, 2, 10), along with a decrease in the gene expression of tumor and metastasis suppressors (i.e., NME1, PNN). An additional proteomic study was performed in the two types of hydrogels and revealed that the level of MMPs (i.e., MMP1, 13) was higher in viscoelastic hydrogels compared to elastic ones. Viscoelasticity could mediate several cell functions and is directly linked to cell spreading ability in coculture. Thus, from these results, it remains difficult to conclude on the actual role of viscoelasticity as opposed to the effects of cell coculture.

In a different study, BE hydrogels were designed for coculture experiments for breast cancer investigation.^173^ As recent studies suggested that breast cancer ECM shows high similarity with wound healing/remodeling ECM,^174,175^ researchers took advantage of the self-healing property of these hydrogels to mimic breast cancer ECM remodeling. In their approach, human pulmonary fibroblasts (CCL151) and human breast cancer cells (MDA MB 231) were separately encapsulated and cultured in two identical BE hydrogels (*G*′ ≈ 1 kPa) composed of PVA, a PBA-containing vinyl copolymer, and fibronectin for cell adhesion. Then, the two hydrogels were put together to recreate a healing environment. The authors confirmed the successful bonding of the two disparate hydrogels with the effective migration of the two cell types across the initial interface. They further observed that both cell types (CCL151 and MDA MB 231) were able to proliferate but had different migration capacities: fibroblasts migrated over short distances but in a large number, while a smaller number of breast cancer cells was able to migrate through the hydrogel but over longer distances. While these are preliminary results, this study provides an interesting proof of concept of the use of BE hydrogels for coculture experiments and the mimicry of biological remodeling.

### 4.3 Boronate Ester Hydrogels for Material-Guided Endogenous Repair

**Figure 4:**
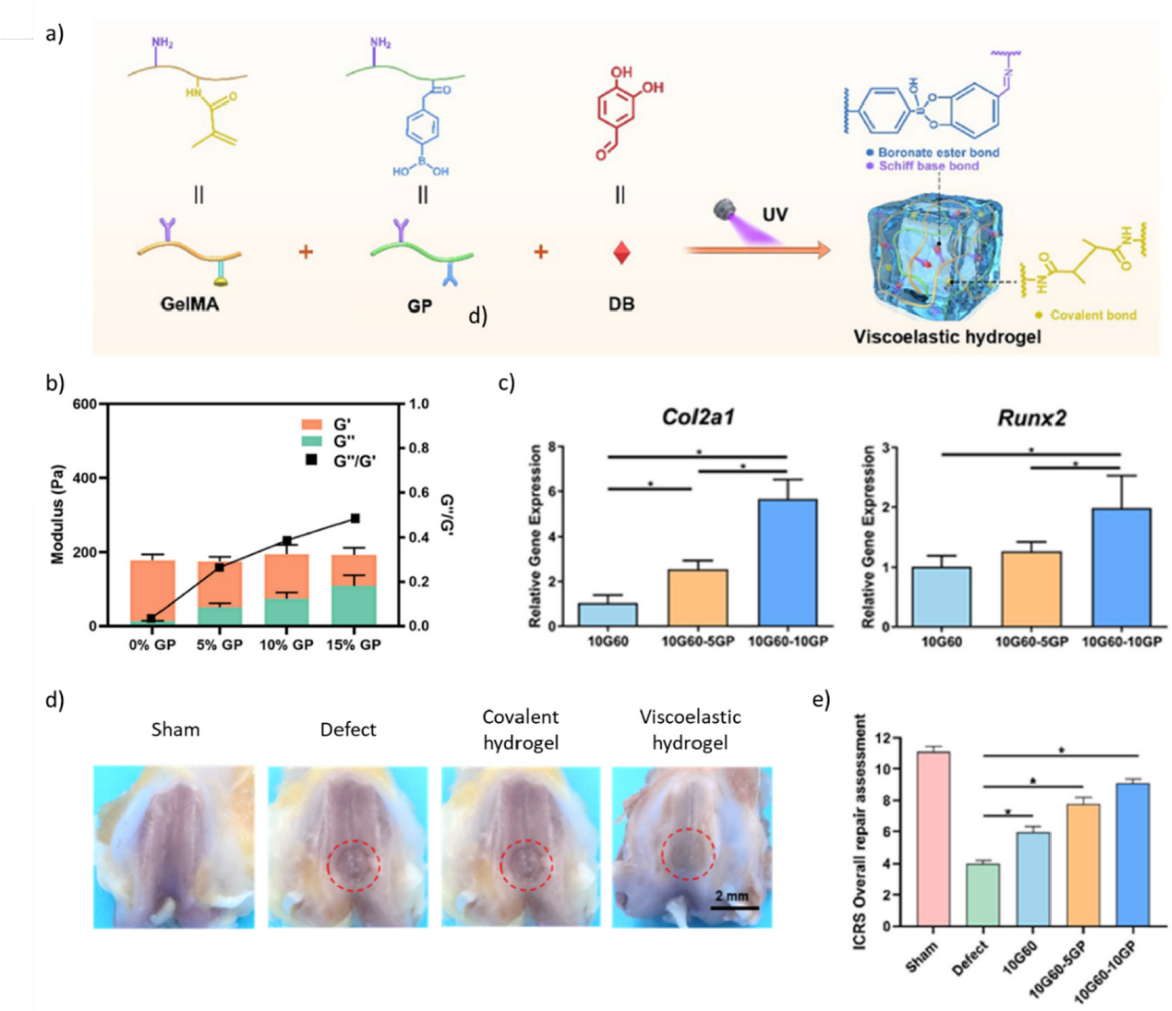
Boronate ester hydrogel for BMSC differentiation and osteochondral defect repair. (a) Schematic of the combination of methacrylated gelatin (GelMA) and PBA-modified gelatin (GP) with 3,4-dihydroxybenzaldehyde cross-linker for the formation of a viscoelastic hydrogel. (b) Storage modulus (*G*′), loss modulus (*G*′’) and the *G*′’/*G*′ ratio of different hydrogel formulations (i.e., concentration of GP). Varying the GP concentration enabled to tune the hydrogel viscoelasticity (*G*′’/*G*′ ratio) while keeping *G*′ constant. (c) Upregulation of the gene expression of chondrogenic (*Col2a1*) and osteogenic (*Runx2*) markers measured by RT-PCR with increasing viscoelasticity. (d) Macroscopic results of the cartilage defect repair after 12 weeks. The red circles show the locations of the defects and highlight a better macroscopic osteochondral defect repair with a viscoelastic hydrogel compared to an elastic hydrogel. e) ICRS cartilage repair macroscopic scores reveal that repair increases with viscoelasticity (adapted with permission from Liu et al.^168^). Copyright [2023][KeAi].

To date, few studies investigated the effect of BE hydrogel implantation on surrounding cells and tissues in vivo. As described above (see section III.b.), Liu et al. demonstrated that BE hydrogel could favor osteogenic and chondrogenic lineages of encapsulated BMSCs.^168^ Using the same hydrogel formulations, the authors hypothesized that a cell-free BE hydrogel could be used for the treatment of osteochondral (OC) defects (Figure 4). Using a rat model with induced femoral OC defect (2 mm in diameter and depth), they compared the effect of the implantation of a covalent hydrogel (i.e., GelMA) and two BE hydrogels with distinct viscoelastic properties (low and high viscoelasticity, corresponding to tan δ of 0.3 and 0.4) to a suture only group after 12 weeks. At a macroscopic scale, increasing viscoelasticity seemed to promote the formation of a cartilaginous tissue similar in aspect ratio to the native surrounding tissue. Using microcomputed tomography, the authors observed a significant increase in bone volume fraction (BVF), suggesting new bone formation, with a BVF of ≈25%, 30%, and 40% for the covalent, low and high viscoelasticity hydrogels, respectively. The tissue repair quality was then evaluated using the International Cartilage Repair Society (ICRS) macroscopic score. For the three groups, the authors reported an ICRS score of 6, 8, and 9, respectively, suggesting that increasing the viscoelasticity strongly improved OC repair. However, the authors did not perform a more detailed characterization of the newly formed tissues (bone and cartilage); therefore, they could not fully conclude on the contribution of endogenous cells to the repair. Further investigations are needed to highlight the mechanisms underlying the endogenous repair process associated with these viscoelastic hydrogels.

For the treatment of brain lesions (i.e., injured or diseased brain), the use of implantable biomaterials is often limited by the fact that surrounding brain cells cannot invade implanted scaffolds, preventing neotissue formation and tissue repair. Thus, the capacity of BE hydrogels to allow for cell invasion owing to bond rearrangement and viscoelasticity has attracted attention for the development of new treatments of brain lesions. In a recent study, a BE hydrogel was designed using PBA/dopamine cross-linking that combined HA, which is a natural brain ECM component, and gelatin, which allows for cell adhesion.^46^ The viscoelastic behavior of the hydrogel (tan δ = 0.33) was optimized to match that of the rat brain parenchyma (tan δ = 0.31). 21 days after implantation in a murine model of traumatic brain injury, the BE hydrogel led to total wound closure, while less than 50% wound closure was observed in the absence of hydrogel injection. The hydrogel also led to a progressive reduction of the glial scar (GFAP marker), which is characterized by ECM deposition right after brain injury and generally hinders neural generation. Finally, the study reported that neurons (MAP2 marker) were able to invade and proliferate in the hydrogel at the wound site. Beyond the hydrogel composition, which may provide a more suitable environment for neural cell growth and migration, the authors hypothesized that the hydrogel viscoelasticity could play an important role in neural tissue development. Of note, the functionality of the newly formed brain tissue was not assessed in this study, calling for further investigation. Together, this work suggests that the highly permissive nature of BE hydrogels could be advantageously used to promote brain repair and, more broadly, be used in wound healing applications. Combining BE hydrogels and molecules associated with wound repair, such as antibacterial or antifungal drugs, also constitutes an interesting avenue of research.

Overall, it is commonly accepted that hydrogels for therapeutic applications should best mimic the composition and physical properties of the natural ECM of the transplanted cells or target tissue. However, the synthesis of fully biomimetic matrices remains difficult. More importantly, the benefits of a biomimetic design are rarely demonstrated. In this context, priority may be given to more systematic studies where hydrogel compositions are screened for a given therapeutic application, and BE hydrogels with more tunable properties could be a key to success.

### 4.4 Boronate Ester Hydrogels in Bioprinting

3D bioprinting is increasingly used for biomedical applications.^176,177^ Among the various bioprinting technologies,^178^ extrusion-based bioprinting is the most commonly used because it is relatively simple and affordable. However, designing extrudable materials that allow for high printing resolution (filament <200 μm in diameter) and good shape fidelity while being tunable in terms of polymer composition and mechanical properties remains a challenge. To succeed, a variety of strategies have been proposed to control the transition from a liquid in the printing nozzle to a solid on the printing platform, mostly relying on the use of photo-cross-linking^179−181^ and/or support bath.^182−184^ As a complementary approach, dynamic covalent hydrogels, including BE hydrogels, have been investigated as potential printable materials based on their ability to flow after cross-linking.^185−187^

#### 4.4.1 Double Network BE Hydrogels As Bioinks

BE hydrogels can easily flow and are therefore extrudable. However, their fast relaxation and lack of stability in culture medium usually do not allow them to maintain a predefined shape for extended periods of time. To take advantage of their extrudability, BE hydrogels have been combined with additional cross-linking strategies that allow for postprinting stabilization.^188^ For example, a study reported the association of alginate, which contains diol groups, with PBA-modified laminarin (a polymer extracted from brown algae) to produce a BE hydrogel that can be stabilized by ionic cross-linking with calcium ions.^189^ For 3D printing, each printed hydrogel layer was sprayed with CaCl_2_ allowing the authors to print an 8-layer construct (3.2 mm height, 10 mm diameter) without structural collapse. The cytocompatibility of the approach was then validated using three different cell lines MC3T3-E1 (osteoblast precursors), L929 (fibroblast), and MDA (breast carcinoma), which were all homogeneously distributed throughout the printed constructs and remained viable (>90%) for at least 2 weeks. Although promising, the spraying step after the printing of each layer made the process relatively difficult to implement and extended printing time. More importantly, alginate was previously shown not to bind PBA effectively at physiological pH,^58^ raising the question of the true contribution of BE cross-linking to this system, which could in principle be replaced by an alginate solution that is viscous enough to be printed.

In a different approach, BE cross-linking was combined with slow covalent cross-linking for single-step bioprinting. Here, the BE hydrogel was expected to be printable over a defined period of time before spontaneously cross-linking via covalent bonds for construct stabilization. To succeed, the authors used a BE hydrogel composed of PVA and PBA-modified HA. The HA component was further modified with acrylate groups so that the BE hydrogel would covalently cross-link when mixed with thiolated gelatin within 1 h at 37 °C.^190^ This dually cross-linked hydrogel was stable over 15 days before slow degradation (50% mass loss after 30 days), allowing for cell culture experiments. The authors demonstrated that their BE hydrogel had antioxidative properties owing to the presence of PBA moieties. In response to an H_2_O_2_ treatment_,_ mouse chondrocytes encapsulated in standard alginate hydrogel beads had downregulated expression of *ACAN* and *COL2*, two ECM genes associated with anabolism, as well as upregulated expression of *MMP13*, a catabolic ECM gene. Conversely, the expression of these three genes did not significantly change when chondrocytes were encapsulated in the BE hydrogel. Together, with the increase in glycosaminoglycan (GAG) concentration over 2 weeks of culture, these results suggest that this printable hydrogel maintained the encapsulated chondrocyte phenotype, paving the way for cartilaginous tissue bioprinting. Despite relevant biological results, the printing resolution remained limited, with an optimal filament diameter of 860 μm obtained from a 30G nozzle (inner diameter of 310 μm). More importantly, this approach imposes a printing time window after which the hydrogel will clog the nozzle. Progressive covalent cross-linking also leads to modifications of the bioink physicochemical properties during the printing process, which may require continuous adjustment of the printing parameters–one of the reasons why the entire field has been avoiding the direct printing of covalent hydrogels.

#### 4.4.2 Toward 4D Bioprinting with BE Hydrogels

**Figure 5:**
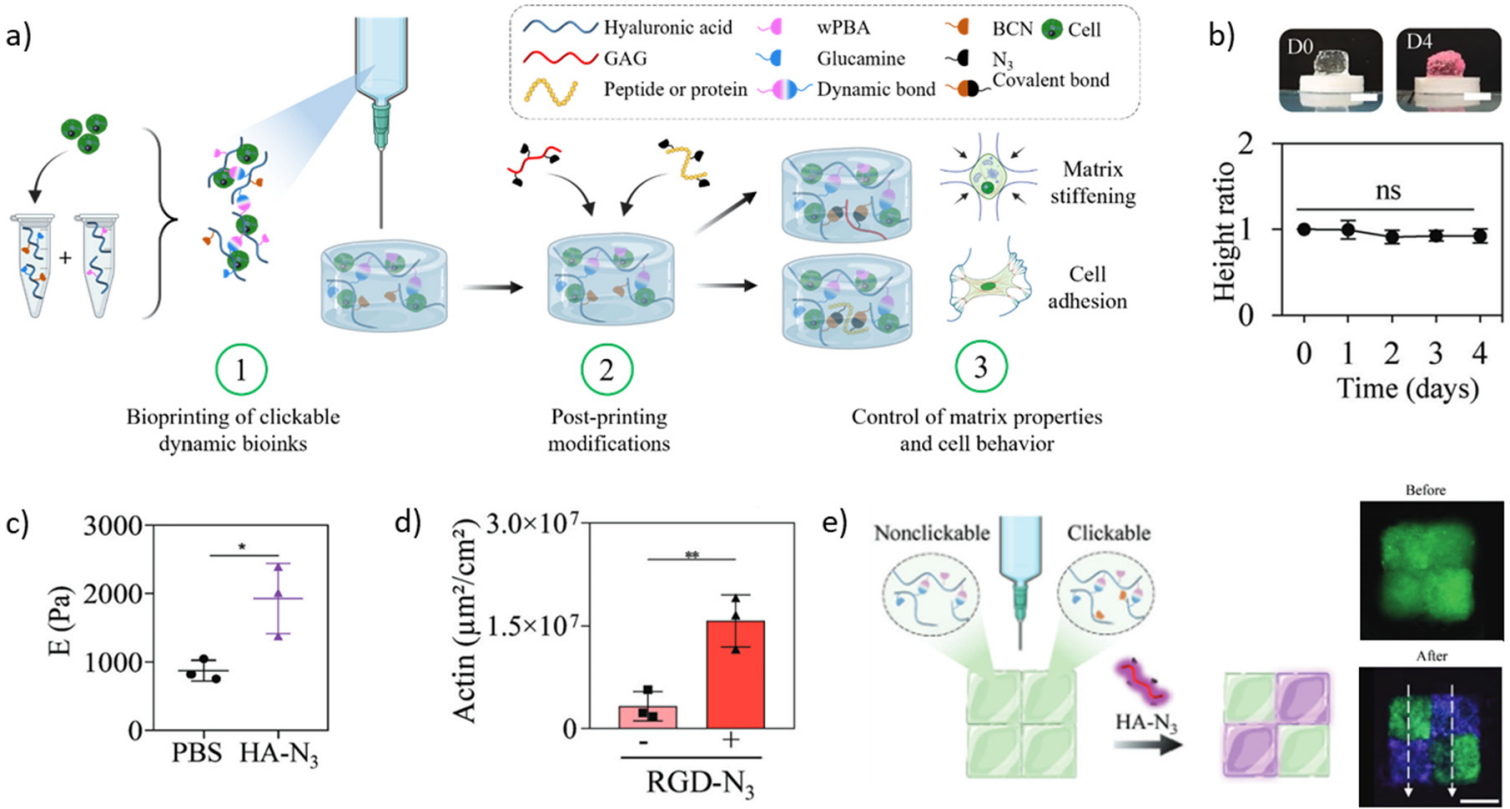
Boronate ester hydrogels for the design of clickable dynamic bioinks. (a) Schematic of the concept of clickable dynamic bioink with BE hydrogel (*o*-AM-PBA- and glucamine-modified HA) modified with bicyclononyne (BCN) moieties to allow postprinting modifications with N_3_-modified molecules through SPAAC click reaction. (b) Pictures and height ratio (normalized to day 0) of the 3D printed cylinders on day 0 and after 4 days of immersion in culture medium, which show no collapse of the structure. (c) Stiffness increase after 24h of immersion in PBS containing HA-N_3_ (purple) or not (black), showing the effective postprinting mechanical reinforcement of the construct. (d) ASC adhesion with or without addition of RGD-N_3_, showing a significant increase in cell adhesion on the top of the hydrogel with clickable RGD. (e) Schematic and pictures of nonclickable and clickable dynamic hydrogels printed side-by-side, before and after 24-h incubation with CF647-HA-N_3_ (purple), demonstrating the possibility of spatial control over the postprinting modifications (adapted with permission from Tournier et al.^191^). Copyright [2023] [John Wiley & Sons].

Recently, we investigated the use of a new class of BE hydrogels that shows long-term stability^54^ as potential biomaterial inks for 3D extrusion-based printing (Figure 5).^191^ Using *o*-AM-PBA/glucamine cross-linking, we optimized two HA-based hydrogel formulations with a *G*′_1 Hz_ of 200 and 2000 Pa, respectively, that could be printed but did not readily collapse under their own weight. The preformed hydrogels prevented cell sedimentation in the cartridge and, thus, ensured homogeneous cell distribution. We showed that the two hydrogels were stable for days in culture medium. We then explored the combination of these cytocompatible hydrogels with a click and bioorthogonal reaction (i.e., SPAAC) for postprinting tunability of the physical and biochemical properties of printed objects. More precisely, we sought to modify our hydrogels with a clickable moiety, so that the properties of a printed object could be adjusted by adding a molecule of interest modified with the complementary clickable moiety to the culture medium. We showed that our clickable BE hydrogels allowed for adjusting the polymer composition (e.g., HA, gelatin) and stiffness (i.e., 2-fold increase) of printed objects.

We further demonstrated that cell adhesion within printed hydrogels can be tuned by adding a clickable adhesive peptide (RGD) to the culture medium. More importantly, we showed that these modifications could be controlled over time and space, paving the way for 4D bioprinting applications. Our study also revealed some limitations for the use of our BE hydrogels as printable biomaterials. First, the instantaneous gelation of BE hydrogels can affect the ability to mix large volumes of hydrogel precursors properly, which can lead to hydrogel heterogeneity and printing difficulties. This may be addressed in the future by the use of light-triggered BE cross-linking. Second, the dynamic nature of BE hydrogels makes them prone to fast coalescence, which does not qualify them for the printing of objects with high resolution or hollow structures. While the development of BE hydrogels with slower relaxation times could improve the printing resolution, it would most likely affect injectability, thus making extrusion difficult.^192^

#### 4.4.3 BE Hydrogels as Sacrificial Materials

The stimuli-responsive properties of BE hydrogels also make them interesting sacrificial materials for the 3D printing of hollow structures. Here, the concept is to print a BE hydrogel filament and cover it with a nonsacrificial material before dissolving the BE hydrogel by either a change in pH or the addition of a competitive molecule (i.e., glucose) to form a hollow structure. For example, Tseng et al. have developed vascular-like channels from BE hydrogels using glucose to trigger dissolution.^76^ The authors extruded a tubular BE hydrogel, which was composed of PEG-diacrylate, dithiothreitol, and borax as a cross-linker, between two layers of nonsacrificial chitosan-based hydrogel. Immersing the construct in conventional high-glucose (4.5 g/L) cell culture medium allowed them to dissolve the BE hydrogel and successfully form channels (≈ 2.5 mm in diameter). To improve the filament resolution and get closer to the diameter of arterioles (≈10–150 μm), the authors designed another BE hydrogel that combined cellulose nanofibrils (CNF) for improved printability, PVA as a diol-containing polymer, and a PBA-containing vinyl copolymer.^193^ This hydrogel was successfully extruded through a 27 G nozzle to form microfilaments with a diameter of 250 μm. The microfilaments were covered with liquid GelMA before UV exposure. Upon the addition of high glucose DMEM medium, the sacrificial BE hydrogel was removed within 5 min at 37 °C, successfully forming microchannels. These microchannels could be perfused with endothelial cells that adhered to the formed lumens and proliferated to mimic the structure of vascular channels.

## 5 Conclusion

BE hydrogels hold great potential as scaffolds for a variety of biomedical applications. Substantial progress has been made to produce BE hydrogels at physiological pH, including the development and use of PBAs with lower p*K*_a, acid_ along with diols with increased reactivity. BE hydrogels have also already been proven useful for the development of injectable and stimuli-responsive therapies, among others. However, more work on BE chemistry is expected to provide the community with BE hydrogels that are stable long-term and tunable in terms of composition, relaxation time, and rigidity. Success will depend on our ability to develop a more multidisciplinary scientific network to tackle this challenge, gathering experts in computational chemistry, organic chemistry, rheology, physical chemistry, biomaterials science, and beyond.

## Supporting Information Available

A table presenting the structures of PBAs and diols that could be used for innovative BE hydrogel design, along with associated references (PDF)

## Footnotes

1 K_eq_ for acethydrazide, benzhydrazide and 6 aminoxyhexanoic acid were calculated from K_d_ values provided in ref (103).
