# Supplementary material for: Boronate Ester Hydrogels for Biomedical Applications: Challenges and Opportunities
Source: Chem Mater. 2024 Jul 9;36(14):6674–95. doi: 10.1021/acs.chemmater.4c00507 (PMC11270748; doi:10.1021/acs.chemmater.4c00507)
Supplement: Supplementary file 1 — cm4c00507_si_001.pdf [file cm4c00507_si_001.pdf]

Supporting Information

Boronate Ester Hydrogels for Biomedical Applications: Challenges and Opportunities

Léa Terriac,[1] Jean-Jacques Helesbeux,[2] Yves Maugars,[1] Jérôme Guicheux,[1] Mark W. Tibbitt,[3] Vianney Delplace[1]*

[1]Nantes Université, Oniris, CHU Nantes, INSERM, Regenerative Medicine and Skeleton, RMeS, UMR 1229, F-44000 Nantes, France

[2]Univ Angers, SONAS, SFR QUASAV, F-49000 Angers, France

[3]Macromolecular Engineering Laboratory, Department of Mechanical and Process Engineering, ETH Zurich, 8092 Zurich, Switzerland

Table S1- Potential PBAs and diols for innovative BE hydrogel design.

	PBA	Primary amine containing molecule	Diol	Product	Reversibility	Ref.
	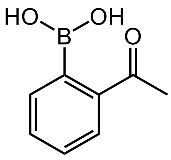	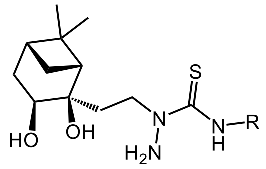		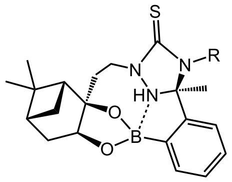	none	1
Iminoboronate ester formation	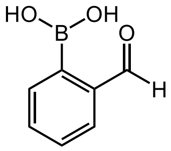	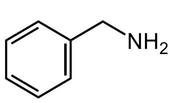	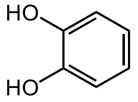	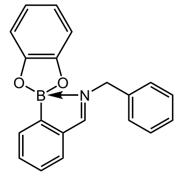	Low reversibility<br>$K_{eq,\ iminoboro.} = 1100\ M^{-1}$<br>$K_{eq,\ BE} = 2.45 \times 10^{3}\ M^{-1}$	2
		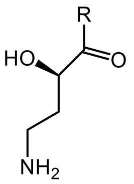	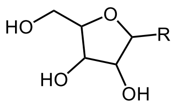	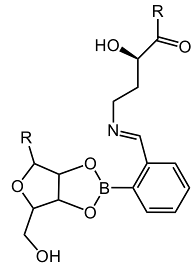	Not measured but mentioned and implied through glucose sensitivity	3
Boronate esters using salicylic acid derivatives	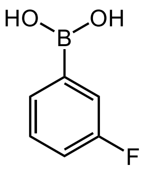	–	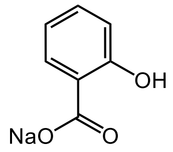	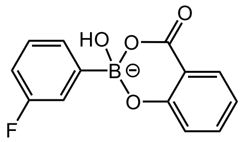	K_{eq}=166 M^{-1}	4
	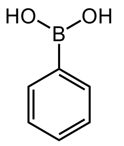	–	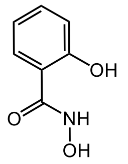	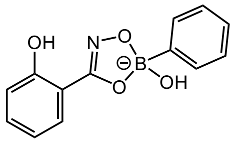<br>or<br>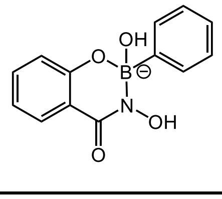	Low reversibility<br>K_{eq}=1.6 x 10^{4} M^{-1}	5

References

(1) Palvai, S.; Bhangu, J.; Akgun, B.; Moody, C. T.; Hall, D. G.; Brudno, Y. In Vivo Targeting Using Arylboronate/Nopoldiol Click Conjugation. *Bioconjugate Chemistry* **2020**, *31* (10), 2288–2292. https://doi.org/10.1021/acs.bioconjchem.0c00453.

(2) Chapin, B. M.; Metola, P.; Lynch, V. M.; Stanton, J. F.; James, T. D.; Anslyn, E. V. Structural and Thermodynamic Analysis of a Three-Component Assembly Forming *Ortho*-Iminophenylboronate Esters. *The Journal of Organic Chemistry* **2016**, *81* (18), 8319–8330. https://doi.org/10.1021/acs.joc.6b01495.

(3) Hu, J.; Hu, Q.; He, X.; Liu, C.; Kong, Y.; Cheng, Y.; Zhang, Y. Stimuli-Responsive Hydrogels with Antibacterial Activity Assembled from Guanosine, Aminoglycoside, and a Bifunctional Anchor. *Advanced Healthcare Materials* **2020**, *9* (2), 1901329. https://doi.org/10.1002/adhm.201901329.

(4) Miki, R.; Yamaki, T.; Uchida, M.; Natsume, H. Diol Responsive Viscosity Increase in a Cetyltrimethylammonium Bromide/Sodium Salicylate/3-Fluorophenylboronic Acid Micelle System. *RSC Advances* **2022**, *12* (11), 6668–6675. https://doi.org/10.1039/D1RA08831A.

(5) Martínez-Aguirre, M. A.; Flores-Alamo, M.; Yatsimirsky, A. K. Thermodynamic and Structural Study of Complexation of Phenylboronic Acid with Salicylhydroxamic Acid and Related Ligands. *Applied Organometallic Chemistry* **2018**, *32* (8). https://doi.org/10.1002/aoc.4405.
